# Large-Scale Simulations of Plastic Neural Networks on Neuromorphic Hardware

**DOI:** 10.3389/fnana.2016.00037

**Published:** 2016-04-07

**Authors:** James C. Knight, Philip J. Tully, Bernhard A. Kaplan, Anders Lansner, Steve B. Furber

**Affiliations:** ^1^Advanced Processor Technologies Group, School of Computer Science, University of ManchesterManchester, UK; ^2^Department of Computational Biology, Royal Institute of TechnologyStockholm, Sweden; ^3^Stockholm Brain Institute, Karolinska InstituteStockholm, Sweden; ^4^Institute for Adaptive and Neural Computation, School of Informatics, University of EdinburghEdinburgh, UK; ^5^Department of Visualization and Data Analysis, Zuse Institute BerlinBerlin, Germany; ^6^Department of Numerical analysis and Computer Science, Stockholm UniversityStockholm, Sweden

**Keywords:** SpiNNaker, learning, plasticity, digital neuromorphic hardware, Bayesian confidence propagation neural network (BCPNN), event-driven simulation, fixed-point accuracy

## Abstract

SpiNNaker is a digital, neuromorphic architecture designed for simulating large-scale spiking neural networks at speeds close to biological real-time. Rather than using bespoke analog or digital hardware, the basic computational unit of a SpiNNaker system is a general-purpose ARM processor, allowing it to be programmed to simulate a wide variety of neuron and synapse models. This flexibility is particularly valuable in the study of biological plasticity phenomena. A recently proposed learning rule based on the Bayesian Confidence Propagation Neural Network (BCPNN) paradigm offers a generic framework for modeling the interaction of different plasticity mechanisms using spiking neurons. However, it can be computationally expensive to simulate large networks with BCPNN learning since it requires multiple state variables for each synapse, each of which needs to be updated every simulation time-step. We discuss the trade-offs in efficiency and accuracy involved in developing an event-based BCPNN implementation for SpiNNaker based on an analytical solution to the BCPNN equations, and detail the steps taken to fit this within the limited computational and memory resources of the SpiNNaker architecture. We demonstrate this learning rule by learning temporal sequences of neural activity within a recurrent attractor network which we simulate at scales of up to 2.0 × 104 neurons and 5.1 × 107 plastic synapses: the largest plastic neural network ever to be simulated on neuromorphic hardware. We also run a comparable simulation on a Cray XC-30 supercomputer system and find that, if it is to match the run-time of our SpiNNaker simulation, the super computer system uses approximately 45× more power. This suggests that cheaper, more power efficient neuromorphic systems are becoming useful discovery tools in the study of plasticity in large-scale brain models.

## 1. Introduction

Motor, sensory and memory tasks are composed of sequential elements and are therefore thought to rely upon the generation of temporal sequences of neural activity (Abeles et al., 1995; Seidemann et al., 1996; Jones et al., 2007). However it remains a major challenge to learn such functionally meaningful dynamics within large-scale models using biologically plausible synaptic and neural plasticity mechanisms. Using SpiNNaker, a neuromorphic hardware platform for simulating large-scale spiking neural networks, and BCPNN, a plasticity model based on Bayesian inference, we demonstrate how temporal sequence learning could be achieved through modification of recurrent cortical connectivity and intrinsic excitability in an attractor memory network.

Spike-Timing-Dependent Plasticity (Bi and Poo, 1998) (STDP) inherently reinforces temporal causality which has made it a popular choice for modeling temporal sequence learning (Dan and Poo, 2004; Caporale and Dan, 2008; Markram et al., 2011). However, to date, all large-scale neural simulations using STDP (Morrison et al., 2007; Kunkel et al., 2011) have been run on large cluster machines or supercomputers, both of which consume many orders of magnitude more power than the few watts required by the human brain. Mead (1990) suggested that the solution to this huge gap in power efficiency was to develop an entirely new breed of “neuromorphic” computer architectures inspired by the brain. Over the proceeding years, a number of these neuromorphic architectures have been built with the aim of reducing the power consumption and execution time of large neural simulations.

Large-scale neuromorphic systems have been constructed using a number of approaches: NeuroGrid (Benjamin et al., 2014) and BrainScaleS (Schemmel et al., 2010) are built using custom analog hardware; True North (Merolla et al., 2014) is built using custom digital hardware and SpiNNaker (Furber et al., 2014) is built from software programmable ARM processors.

Neuromorphic architectures based around custom hardware, especially the type of sub-threshold analog systems which Mead (1990) proposed, have huge potential to enable truly low-power neural simulation, but inevitably the act of casting algorithms into hardware requires some restrictions to be accepted in terms of connectivity, learning rules, and control over parameter values. As an example of these restrictions, of the large-scale systems previously mentioned, only BrainScaleS supports synaptic plasticity in any form implementing both short-term plasticity and pair-based STDP using a dedicated mixed-mode circuit.

As a software programmable system, SpiNNaker will require more power than a custom hardware based system to simulate a model of a given size (Stromatias et al., 2013). However this software programmability gives SpiNNaker considerable flexibility in terms of the connectivity, learning rules, and ranges of parameter values that it can support. The neurons and synapses which make up a model can be freely distributed between the cores of a SpiNNaker system until they fit within memory; and the CPU and communication overheads taken in advancing the simulation can be handled within a single simulation time step.

This flexibility has allowed the SpiNNaker system to be used for the simulation of large-scale cortical models with up to 5.0 × 104 neurons and 5.0 × 107 synapses (Sharp et al., 2012, 2014); and various forms of synaptic plasticity (Jin et al., 2010; Diehl and Cook, 2014; Galluppi et al., 2015; Lagorce et al., 2015). In the most recent of these papers, Galluppi et al. (2015) and Lagorce et al. (2015) demonstrated that Sheik et al.'s (2012) model of the learning of temporal sequences from audio data can be implemented on SpiNNaker using a voltage-gated STDP rule. However, this model only uses a small number of neurons and Kunkel et al.'s (2011) analysis suggests that STDP alone cannot maintain the multiple, interconnected stable attractors that would allow spatio-temporal sequences to be learnt within more realistic, larger networks. This conclusion adds to growing criticism of simple STDP rules regarding their failure to generalize over experimental observations (see e.g., Lisman and Spruston, 2005, 2010; Feldman, 2012 for reviews).

We address some of these issues by implementing spike-based BCPNN (Tully et al., 2014)—an alternative to phenomenological plasticity rules which exhibits a diverse range of mechanisms including Hebbian, neuromodulated, and intrinsic plasticity—all of which emerge from a network-level model of probabilistic inference (Lansner and Ekeberg, 1989; Lansner and Holst, 1996). BCPNN can translate correlations at different timescales into connectivity patterns through the use of locally stored synaptic traces, enabling a functionally powerful framework to study the relationship between structure and function within cortical circuits. In Sections 2.1–2.3, we describe how this learning rule can be combined with a simple point neuron model as the basis of a simplified version of Lundqvist et al.'s (2006) cortical attractor memory model. In Sections 2.4, 2.5, we then describe how this model can be simulated efficiently on SpiNNaker using an approach based on a recently proposed event-driven implementation of BCPNN (Vogginger et al., 2015). We then compare the accuracy of our new BCPNN implementation with previous non-spiking implementations (Sandberg et al., 2002) and demonstrate how the attractor memory network can be used to learn and replay spatio-temporal sequences (Abbott and Blum, 1996). Finally, in Section 3.3, we show how an anticipatory response to this replay behavior can be decoded from the neurons' sub-threshold behavior which can in turn be used to infer network connectivity.

## 2. Materials and methods

### 2.1. Simplified cortical microcircuit architecture

We constructed a network using connectivity based on a previously proposed cortical microcircuit model (Lundqvist et al., 2006) and inspired by the columnar structure of neocortex (Mountcastle, 1997). The network consists of *N*_*HC*_ hypercolumns arranged in a grid where each hypercolumn consists of 250 inhibitory basket cells and 1000 excitatory pyramidal cells evenly divided into 10 minicolumns. Within each hypercolumn, the pyramidal cells send AMPA-mediated connections to the basket cells with a connection probability of 10 % and a weight of 0.4 nA (defined as a postsynaptic current (PSC) amplitude). The basket cells then send GABAergic connections back to the pyramidal cells with a connection probability of 10 % and a weight of 2 nA. The basket cells are also recurrently connected through GABAergic connections, again with a connection probability of 10 % and a connection weight of 2 nA. The functional outcome of this local connectivity (excitatory to inhibitory and vice versa) is to enable winner-take-all (WTA) dynamics within each hypercolumn. While the strength of the local synapses remains fixed, all pyramidal cells in the network are also recurrently connected to each other through global AMPA and NMDA connections using plastic BCPNN synapses (see Section 2.2): also with a connection probability of 10 %. All connections in the network have distance-dependent synaptic delays such that, between two cells located in hypercolumns $H_{xy}^{pre}$ and $H_{xy}^{post}$, the delay is calculated based on the Euclidean distance between the grid coordinates of the hypercolumns (meaning that all local connections have delays of 1 ms):

(1) $$\begin{array}{ll} t_{d}^{H_{xy}^{pre}H_{xy}^{post}}=\frac{d_{norm}\sqrt{{\left(H_{x}^{post}-H_{x}^{pre}\right)}^{2}+{\left(H_{y}^{post}-H_{y}^{pre}\right)}^{2}}}{V}+1 & \; \end{array}$$

Where conduction velocity *V* = 0.2 mm ms^−1^ and *d_norm_* = 0.75 mm.

### 2.2. Synaptic and intrinsic plasticity model

The spike-based BCPNN learning rule is used to learn the strengths of all global synaptic connections and the intrinsic excitabilities of all pyramidal cells in the network described in Section 2.1. The goal of the learning process is to estimate the probabilities of pre- and postsynaptic neurons firing (*P*_*i*_ and *P*_*j*_ respectively), along with the probability of them firing together (*P*_*ij*_). Then, as Lansner and Holst (1996) describe, these probabilities can be used to calculate the synaptic strengths and intrinsic excitabilities of the network allowing it to perform Bayesian inference. Tully et al. (2014) developed an approach for estimating these probabilities based on pre- and postsynaptic spike trains (*S*_*i*_ and *S*_*j*_ respectively), defined as summed Dirac delta functions δ(·) where $t_{i,j}^{f}$ represent the times of spikes:

(2) $$\begin{array}{lllll} S_{i}\left(t\right)=\underset{t_{i}^{f}}{\sum }\delta \left(t-t_{i}^{f}\right) & \; & S_{j}\left(t\right)=\underset{t_{j}^{f}}{\sum }\delta \left(t-t_{j}^{f}\right) & \; & \; \end{array}$$

These spike trains are then smoothed using exponentially weighted moving averages to calculate the *Z* traces:

(3) $$\begin{array}{lllll} \tau_{z_{i}}\frac{dZ_{i}}{dt}=\frac{S_{i}}{f_{max}\Delta t}-Z_{i} & \; & \tau_{z_{j}}\frac{dZ_{j}}{dt}=\frac{S_{j}}{f_{max}\Delta t}-Z_{j} & \; & \; \end{array}$$

Here, the maximum allowed firing rate *f*_*max*_ and spike duration Δ*t* = 1 ms combine with the lowest attainable probability estimate $\epsilon =\frac{1000}{f_{max}\tau_{p}}$ introduced in Equation (5) to maintain a linear mapping from neuronal spike rates to probabilities. For more details on the Bayesian transformation entailed by these equations, see Tully et al. (2014). The *Z* trace time constants τ_*z*_*i*__ and τ_*z*_*j*__ determine the time scale over which correlations can be detected and are inspired by fast biological processes such as Ca^2+^ influx via NMDA receptors or voltage-gated Ca^2+^ channels. The *Z* traces are then fed into the *P* traces, where a coactivity term is introduced:

(4) $$\begin{array}{llllllll} \tau_{p}\frac{dP_{i}}{dt}=Z_{i}-P_{i} & \; & \tau_{p}\frac{dP_{ij}}{dt}=Z_{i}Z_{j}-P_{ij} & \; & \tau_{p}\frac{dP_{j}}{dt}=Z_{j}-P_{j} & \; & \; & \; \end{array}$$

The *P* trace time constant τ_*p*_ models long-term memory storage events such as gene expression or protein synthesis. It can be set higher to more realistically match these slow processes, but since simulation time increases with higher τ_*p*_ values, in this work we keep them just long enough to preserve the relevant dynamics. Estimated levels of activity in the *P* traces are then combined to compute a postsynaptic bias membrane current *I*_β_*j*__ and synaptic weight between pre- and postsynaptic neurons *w*_*ij*_:

(5) $$\begin{array}{lllll} I_{\beta_{j}}=\beta_{gain}\log\left(P_{j}+\epsilon \right) & \; & w_{ij}=w_{gain}^{syn}\log\frac{P_{ij}+\epsilon^{2}}{\left(P_{i}+\epsilon \right)\left(P_{j}+\epsilon \right)} & \; & \; \end{array}$$

Here, β_*gain*_ is used to scale the BCPNN bias into an intrinsic input current to the neuron which is used to model an A-type K+ channel (Jung and Hoffman, 2009) or other channel capable of modifying the intrinsic excitability of a neuron (Daoudal and Debanne, 2003). Similarly, $w_{gain}^{syn}$ is used to scale the BCPNN weight into a current-based synaptic strength.

### 2.3. Neuronal model

We model excitatory and inhibitory cells as IAF neurons with exponentially decaying PSCs (Liu and Wang, 2001; Rauch et al., 2003). The sub-threshold membrane voltage *V*_*m*_ of these neurons evolves according to:

(6) $$\begin{array}{lll} \tau_{m}\frac{dV_{m}}{dt} & =-V_{m}+R_{m}\left(I_{s}+I_{a}+I_{\beta_{j}}\right)\; & \; \end{array}$$

The membrane time constant τ_*m*_ and capacitance *C*_*m*_ determine the input resistance $R_{m}=\frac{\tau_{m}}{C_{m}}$ through which input currents from the afferent synapses (*I*_*s*_), spike-frequency adaption mechanism (*I*_*a*_) and the intrinsic input current from the BCPNN learning rule (*I*_β_*j*__) – described in Section 2.2—are applied. When *V*_*m*_ reaches the threshold *V*_*t*_ a spike is emitted, *V*_*m*_ is reset to *V*_*r*_ and α is added to the adaption current *I*_*a*_. We use Liu and Wang's (2001) model of spike-frequency adaption with the adaption time constant τ_*a*_:

(7) $$\begin{array}{lll} \tau_{a}\frac{dI_{a}}{dt} & =-I_{a}\; & \; \end{array}$$

The synaptic input current to postsynaptic neuron *j* (*I*_*s*_*j*__) is modeled as a sum of exponentially shaped PSCs from other presynaptic neurons in the network:

(8) $$\begin{array}{ll} \tau_{syn}\frac{dI_{s_{j}}}{dt}=-I_{s_{j}}+\underset{syn}{\sum }\overset{n}{\underset{i=0}{\sum }}w_{ij}^{syn}\underset{t_{i}^{f}}{\sum }\delta \left(t-t_{\;i}^{\;f}\right) & \; \end{array}$$

$w_{ij}^{syn}$ indicates the weight of the connection between neurons *i* and *j* [where *syn* ∈ (AMPA, GABA, NMDA) denotes the synapse type], $t_{i}^{f}$ represents the arrival time of spikes from presynaptic neuron *i* (where there are *n* neurons in the network), and τ_*syn*_ is the synaptic time constant.

### 2.4. Simulating spiking neural networks on spinnaker

**Figure 1:**
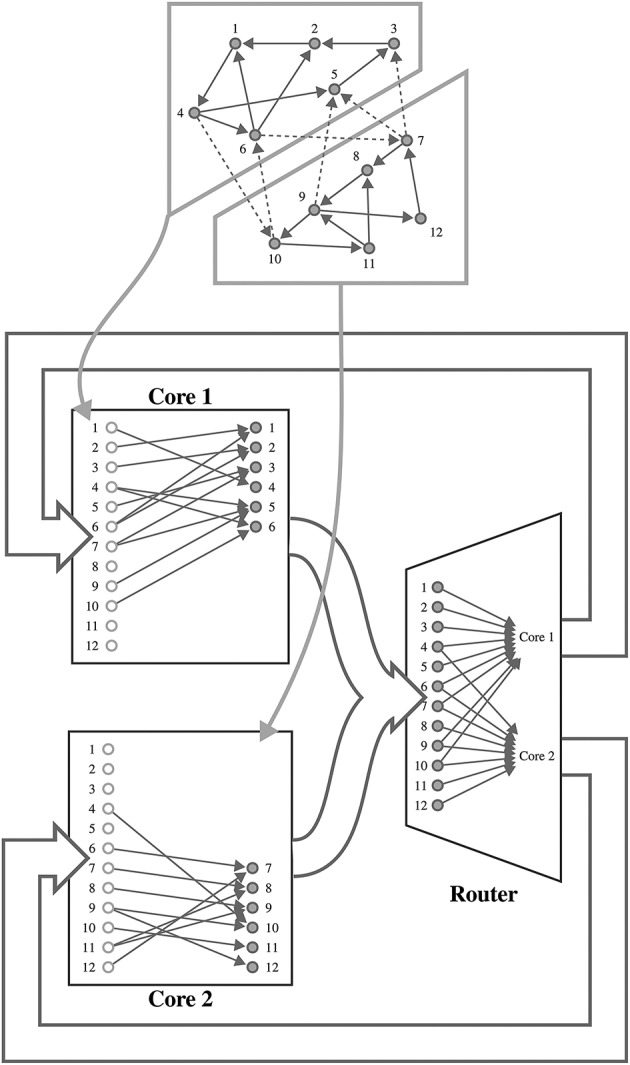
**Mapping of a spiking neural network to SpiNNaker**. For example a network consisting of 12 neurons is distributed between two SpiNNaker cores. Each core is responsible for simulating six neurons (filled circles) and holds a list of afferent synapses (non-filled circles) associated with each neuron in the network. The SpiNNaker router routes spikes from firing neurons (filled circles) to the cores responsible for simulating the neurons to which they make efferent synaptic connections.

SpiNNaker is a digital neuromorphic architecture designed for the simulation of spiking neural networks. Although systems built using this architecture are available in sizes ranging from single boards to room-size machines, they all share the same basic building blocks—the SpiNNaker chip (Furber et al., 2014). Each of these chips is connected to its six immediate neighbors using a chip-level interconnection network with a hexagonal mesh topology. Each SpiNNaker chip contains 18 ARM cores connected to each other through a network-on-chip, and connected to an external network through a multicast router. Each core has two small tightly-coupled memories: 32 KiB for instructions and 64 KiB for data; and shares 128 MiB of off-chip SDRAM with the other cores on the same chip. Although this memory hierarchy is somewhat unusual, the lack of global shared memory means that many of the problems of simulating large spiking neural networks on a SpiNNaker system are shared with more typical distributed computer systems. Morrison et al. (2005) and Kunkel et al. (2012) developed a collection of approaches for mapping such networks onto large distributed systems in a memory-efficient manner while still obtaining supra-linear speed-up as the number of processors increases. The SpiNNaker neural simulation kernel employs a very similar approach where, as shown in Figure 1, each processing core is responsible for simulating between 100 and 1000 neurons and their afferent synapses. The neurons are simulated using a time-driven approach with their state held in the tightly-coupled data memory. Each neuron is assigned a 32 bit ID and, when a simulation step results in a spike, it sends a packet containing this ID to the SpiNNaker router. These “spike” packets are then routed across the network fabric to the cores that are responsible for simulating these synapses. Biological neurons have in the order of 10^3^ – 10^4^ afferent synapses, so updating all of these every time step would be extremely computationally intensive. Instead, as individual synapses only receive spikes at relatively low rates, they can be updated only when they transfer a spike as long as their new state can be calculated from:

1. The synapse's previous state.
2. The time since the last spike was transferred.
3. Information available from the time-driven simulation of the postsynaptic neuron.

Using this event-driven approach on SpiNNaker is also advantageous as, due to their sheer number, synapses need to be stored in the off-chip SDRAM which has insufficient bandwidth for every synapse's parameters to be retrieved every simulation time step (Painkras and Plana, 2013). Instead, on receipt of a “spike” packet, a core retrieves the row of the connectivity matrix associated with the firing neuron from SDRAM. Each of these rows describes the parameters associated with the synapses connecting the firing neuron to those simulated on the core. Once a row is retrieved the weights are inserted into an input ring-buffer, where they remain until any synaptic delay has elapsed and they are applied to the neuronal input current.

In addition to enabling large-scale simulations with static synapses, this event-driven approach can, in theory, be extended to handle any type of plastic synapse that meets the 3 criteria outlined above. However, simulating plastic synapses has additional overheads in terms of memory and CPU load—both of which are very limited resources on SpiNNaker. Several different approaches have been previously developed that aim to minimize memory usage (Jin et al., 2010), reduce CPU load (Diehl and Cook, 2014) or offload the processing of plastic synapses to dedicated cores (Galluppi et al., 2015). Morrison et al. (2007) also extended their work on distributed spiking neural network simulation to include synaptic plasticity, developing an algorithm for simulating plastic synapses in an event-driven manner, using a simplified model of synaptic delay to reduce CPU and memory usage. In this work, we combine elements of Diehl and Cook's (2014) and Morrison et al.'s (2007) approaches, resulting in Algorithm 1 which is called whenever the connectivity matrix row associated with an incoming “spike” packet is retrieved from the SDRAM. As well as the weights of the synapses connecting the presynaptic neuron to the postsynaptic neurons simulated on the local core (*w*_*ij*_), the row also has a header containing the time at which the presynaptic neuron last spiked (*t*_*old*_) and its state at that time (*s*_*i*_). The exact contents of the state depends on the plasticity rule being employed, but as only the times of presynaptic spikes are available at the synapse, the state often consists of one or more low-pass filtered versions of this spike train.

**Algorithm 1:** Algorithmic Implementation of STDP

**function** processRow(*t*)
**for** **each** *j* **in** *postSynapticNeurons* **do**
*history* ← *getHistoryEntries* (*j*, *t_old_*, *t*)

**for** **each** (*t_j_*, *s_j_*) **in** *history* **do**
*w_ij_* ← *applyPostSpike* (*w_ij_*, *t_j_*, *t_old_*, *s_i_*)

(*t_j_*, *s_j_*) ← *getLastHistoryEntry*(*t*)
*w_ij_* ← *applyPreSpike*(*w_ij_*, *t*, *t_j_*, *s_j_*)
*addWeightToRingBuffer*(*w_ij_*, *j*)

*s_i_* ← *addPreSpike*(*s_i_*, *t*, *t_old_*)
*t_old_* ← *t*

The algorithm begins by looping through each postsynaptic neuron (*j*) in the row and retrieving a list of the times (*t*_*j*_) at which that neuron spiked between *t*_*old*_ and *t* and its state at that time (*s*_*j*_). In the SpiNNaker implementation, these times and states are stored in a fixed-length circular queue located in the tightly-coupled data memory to which a new entry gets added whenever a local neuron fires. Next, the effect of the interaction between these postsynaptic spikes and the presynaptic spike that occurred at *t*_*old*_ is applied to the synapse using the *applyPostSpike* function. The synaptic update is then completed by applying the effect of the interaction between the presynaptic spike that instigated the whole process and the most recent postsynaptic spike to the synapse using the *applyPreSpike* function before adding this weight to the input ring buffer. Finally, the header of the row is updated by calling the *addPreSpike* function to update *s*_*i*_ and setting *t*_*old*_ to the current time.

### 2.5. An event-based, SpiNNaker implementation of bayesian learning

Equations (3)–(5) cannot be directly evaluated within the event-driven synaptic processing scheme outlined in Section 2.4, but as they are simple first-order linear ODEs, they can be solved to obtain closed-form solutions for *Z*(*t*) and *P*(*t*). These equations then need only be evaluated when spikes occur. Vogginger et al. (2015) converted this resultant system of equations into a spike-response model (Gerstner and Kistler, 2002) which, as it only consists of linear combinations of $e^{\frac{-t}{\tau_{z}}}$ and $e^{\frac{-t}{\tau_{p}}}$, can be re-framed into a new set of new state variables $Z_{i}^{*}$, $Z_{j}^{*}$, $P_{i}^{*}$, $P_{j}^{*}$, and $P_{ij}^{*}$. These, like the state variables used in many STDP models are simply low-pass filtered versions of the spike-trains and can be evaluated when a spike occurs at time *t*:

(9) $$\begin{array}{lllll} Z_{i}^{*}\left(t\right)=Z_{i}^{*}\left(t^{last}\right)e^{-\frac{\Delta t}{\tau_{z_{i}}}}+S_{i}\left(t\right) & \; & P_{i}^{*}\left(t\right)=P_{i}^{*}\left(t^{last}\right)e^{-\frac{\Delta t}{\tau_{p}}}+S_{i}\left(t\right) & \; & \; \end{array}$$

Z^*^ and P^*^ can now be stored in the pre and postsynaptic state (*s*_*i*_ and *s*_*j*_) and updated in the *addPreSpike* function called from algorithm 1; and when postsynaptic neurons fire. The correlation trace, *P*_*ij*_ can similarly be re-framed in terms of a new state variable:

(10) $$\begin{array}{ll} P_{ij}^{*}\left(t\right)=P_{ij}^{*}\left(t^{last}\right)e^{-\frac{\Delta t}{\tau_{p}}}+S_{i}\left(t\right)Z_{j}^{*}\left(t\right) & \; \end{array}$$

$P_{ij}^{*}$ can now be stored alongside the synaptic weight *w*_*ij*_ in each synapse and evaluated in the *applyPreSpike* and *applyPostSpike* functions called from algorithm 1. The final stage of the event-based implementation is to obtain the *P*_*i*_, *P*_*j*_ and *P*_*ij*_ values required to evaluate (Equation 5) from the new state variables and thus obtain *w*_*ij*_ and β_*j*_.

(11) $$\begin{array}{lll} P_{i}\left(t\right) & =a_{i}\left(Z_{i}^{*}\left(t\right)-P_{i}^{*}\left(t\right)\right)\; & \; \end{array}$$

(12) $$\begin{array}{lll} P_{ij}\left(t\right) & =a_{ij}\left(Z_{i}^{*}\left(t\right)Z_{j}^{*}\left(t\right)-P_{ij}^{*}\left(t\right)\right)\; & \; \end{array}$$

With the following coefficients used for brevity:

(13) $$\begin{array}{rcl} \tau_{z_{ij}} & = & {\left(\frac{1}{\tau_{z_{i}}}+\frac{1}{\tau_{z_{j}}}\right)}^{-1}\;a_{i}=\frac{1}{f_{max}\left(\tau_{z_{i}}-\tau_{p}\right)}\\ a_{ij} & = & \frac{1}{{f_{max}}^{2}\left(\tau_{z_{j}}+\tau_{z_{i}}\right)\left(\tau_{z_{ij}}-\tau_{p}\right)} \end{array}$$

This approach makes implementing spike-based BCPNN on SpiNNaker feasible from an algorithmic point of view, but limitations of the SpiNNaker architecture further complicate the problem. The most fundamental of these limitations is that, as Moise (2012, p. 20) explains, for reasons of silicon area and energy efficiency, SpiNNaker has no hardware floating point unit. While floating point operations can be emulated in software, this comes at a significant performance cost meaning that performance-critical SpiNNaker software needs to instead use fixed-point arithmetic. Hopkins and Furber (2015) discussed the challenges of using fixed-point arithmetic for neural simulation on the SpiNNaker platform in detail but, in the context of this work, there are two main issues of particular importance. Firstly the range of fixed-point numeric representations is static so, to attain maximal accuracy, the optimal representation for storing each state variable must be chosen ahead of time. Vogginger et al. (2015) investigated the use of fixed-point types for BCPNN as a means of saving memory and calculated that, in order to match the accuracy of a time-driven floating point implementation, a fixed-point format with 10 integer and 12 fractional bits would be required. However, not only is the model described in Section 2.2 somewhat different from the reduced modular model considered by Vogginger et al. (2015), but the ARM architecture only allows 8, 16, or 32 bit types to be natively addressed. Therefore, we re-evaluated these calculations for the SpiNNaker implementation and chose to use 16 bit types for two reasons:

1. In order to implement the *getLastHistoryEntry* and *getHistoryEntries* functions used in algorithm 1, each neuron needs to store a history of $Z_{j}^{*}$ and $P_{j}^{*}$ values in the tightly-coupled data memory, therefore minimizing the size of these variables is important.
2. The SpiNNaker CPU cores can perform multiplication operations on signed 16 bit values faster than it can on 32 bit values, allowing more spikes to be transferred each time-step.

Based on a total of 16 bit, the number of bits used for the integer and fractional parts of the fixed-point representation needs to be determined based on the range of the state variables. As all of the Z^*^ and P^*^ state variables are linear sums of exponential spike responses and P^*^ has the largest time constant, it decays slowest meaning that it will reach the highest value. Therefore we can calculate the maximum value which our fixed-point format must be able to represent in order to handle a maximum spike frequency of *f*_*max*_ as follows:

(14) $$\begin{array}{lll} P_{max}^{*} & =\frac{1}{1-e^{-\frac{1}{f_{max}\times \tau_{p}}}}\; & \; \end{array}$$

In order to match the firing rates of pyramidal cells commonly observed in cortex, low values of the maximum firing rate (*f*_*max*_, e.g., 20 or 50 Hz) are often used with the BCPNN model described in Section 2.2. On this basis, by using a signed fixed-point format with 6 integer and 9 fractional bits, if *f*_*max*_ = 20 Hz, traces with τ_*p*_ < 3.17 s can be represented and, if *f*_*max*_ = 50 Hz, traces with τ_*p*_ < 1.27 s can be represented.

The second problem caused by the lack of floating point hardware is that there is no standard means of calculating transcendental functions for fixed-point arithmetic. This means that the exponential and logarithm functions required to implement BCPNN must be implemented by other means. While it is possible to implement approximations of these functions using, for instance a Taylor series, the resultant functions are likely to take in the order of 100 CPU cycles to evaluate (Moise, 2012), making them too slow for use in the context of BCPNN where around ten of these operations will be performed every time a spike is transferred. Another approach is to use pre-calculated lookup tables (LUTs). These are particularly well suited to implementing functions such as $e^{\frac{-t}{\tau }}$ where *t* is discretized to simulation time steps and, for small values of τ, the function decays to 0 after only a small number of table entries. While the log(*x*) function has neither of these ideal properties, *x* can be normalized into the form *x* = *y* × 2^*n*^ : *n* ∈ ℤ, *y* ∈ [1, 2) so a LUT is only required to cover the interval [1, 2) within which log(*x*) is relatively linear.

## 3. Results

### 3.1. Validating BCPNN learning on SpiNNaker with previous implementations

In this section we demonstrate that the implementation of BCPNN we describe in Section 2.5 produces connection weights and intrinsic excitabilities comparable to those learned by previous models. To do this we used the procedure developed by Tully et al. (2014) and the network described in Table 1 to compare two neurons, connected with a BCPNN synapse, modeled using both our spiking BCPNN implementation and as abstract units with simple, exponentially smoothed binary activation patterns (Sandberg et al., 2002). We performed this comparison by presenting the neurons with five patterns of differing relative activations, each repeated for ten consecutive 200 ms trials. Correlated patterns meant both neurons were firing at *f*_*max*_ Hz or ϵ Hz each trial; independent patterns meant uniform sampling of *f*_*max*_ Hz and ϵ Hz patterns for both neurons in each trial; anti-correlated patterns meant one neuron fired at *f*_*max*_ Hz and the other at ϵ Hz or vice-versa in each trial; both muted meant both neurons fired at ϵ Hz in all trials; and post muted meant uniform sampling of presynaptic neuron activity while the postsynaptic neuron fired at ϵ Hz in all trials.

Table 1**Model description of the BCPNN validation network**.

**Table:** 

**(A) Model summary**	
Populations	Presynaptic, postsynaptic, presynaptic input, postsynaptic input
Connectivity	One-to-one
Neuron model	Leaky integrate-and-fire with exponential-shaped synaptic current inputs and spike-frequency adaption (Liu and Wang, 2001)
Synapse model	Current-based with exponential-shaped PSCs
Plasticity	BCPNN AMPA synapses
Input	Externally generated Poisson spike trains
Measurements	Intrinsic bias current and synaptic weights

**Table:** 

**(B) Populations**		
**Name**	**Elements**	**Size**
Presynaptic	Leaky IAF	1
Postsynaptic	Leaky IAF	1
Presynaptic input	External spike source	1
Postsynaptic input	External spike source	1

**Table:** 

**(C) Connectivity**			
**Source**	**Target**	**Pattern**	**Weight**
Presynaptic input	Presynaptic	One-to-one	2 nA
Postsynaptic input	Postsynaptic	One-to-one	2 nA
Presynaptic	Postsynaptic	One-to-one	Plastic

**Table:** 

**(D) Neuron and synapse model**	
Type	Leaky integrate-and-fire with exponential-shaped synaptic current inputs and spike-frequency adaption (Liu and Wang, 2001) as described in Section 2.3
Parameters	τ_*m*_ = 10 ms membrane time constant
	*C*_*m*_ = 250 pF membrane capacitance
	*V*_*t*_ = −55.4 mV threshold voltage
	*V*_*r*_ = −70 mV reset voltage
	α = 0.0 nA adaption current (disabled)
	τ_AMPA_ = 2.5 ms AMPA synapse time constant

**Table:** 

**(E) Plasticity**	
Type	BCPNN AMPA synapses as described in Section 2.2
Parameters	*f*_*max*_ = 50 Hz maximum spiking frequency
	τ_*z*_*i*__ = 10 ms presynaptic primary trace time constant
	τ_*z*_*j*__ = 10 ms postsynaptic primary trace time constant
	τ_*p*_ = 1000 ms probability trace time constant
	$w_{gain}^{syn}=1\;\text{nA}$ weight gain
	β_*gain*_ = 1 nA intrinsic bias gain

**Table:** 

**(F) Input**	
**Type**	**Description**
Externally generated Poisson spike trains	As described in Section 3.1

*After Nordlie et al. (2009)*.

**Figure 2:**
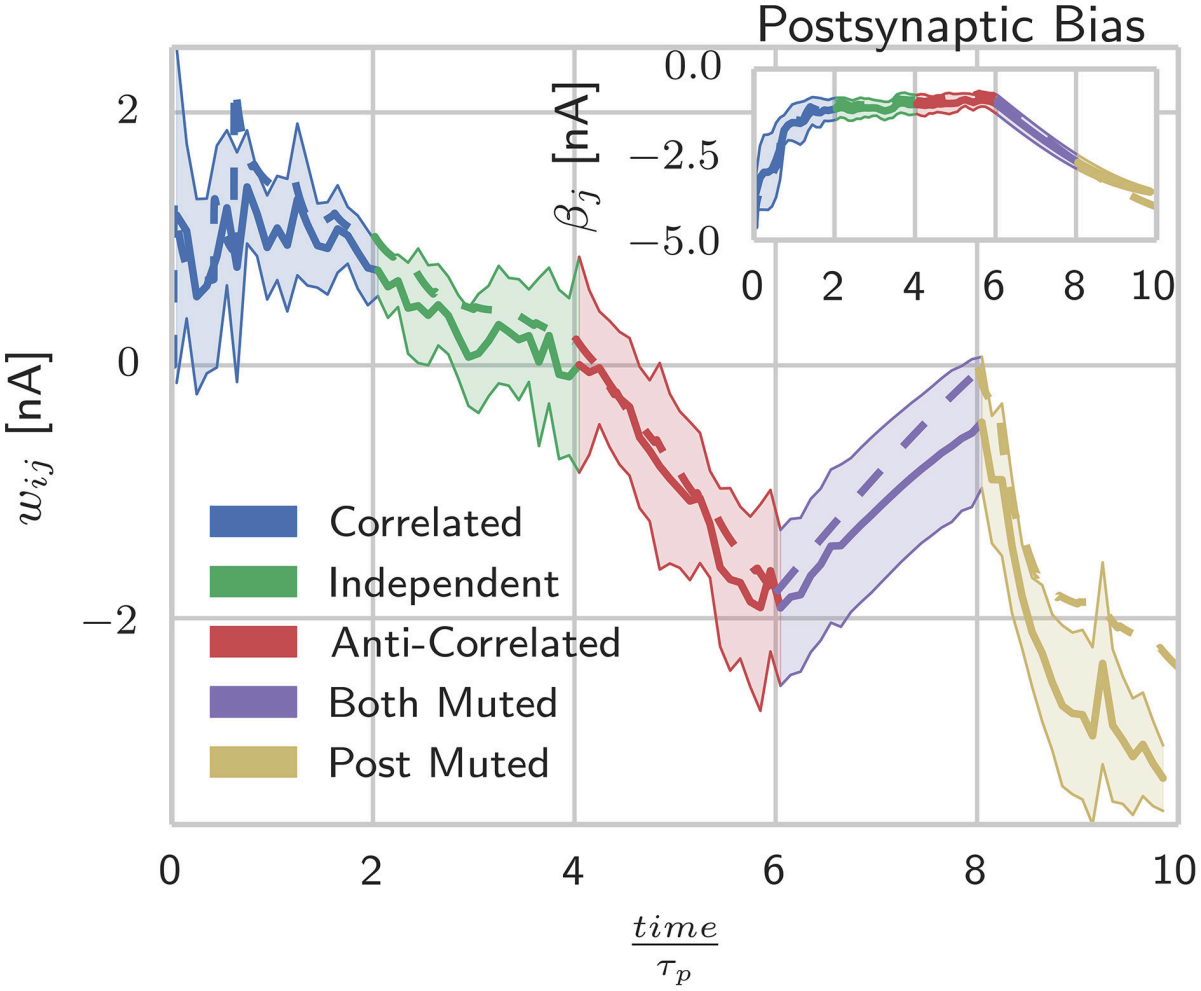
**Spike-based BCPNN estimates abstract BCPNN for different input patterns**. Comparing weight and bias (inset) development under different protocols when using abstract (dotted) and SpiNNaker (solid) versions of the learning rule. SpiNNaker simulations were repeated 10 times and averaged, with standard deviations illustrated by the shaded regions.

As Figure 2 shows, during the presentation of patterns in which both units are firing, the responses from the abstract model fall well within the standard deviation of the SpiNNaker model's responses, but as units are muted, the two models begin to diverge. Further investigation into the behavior of the individual state variables shows that this is due to the P^*^ term of Equation (11) coming close to underflowing the 16 bit fixed-point format when a long time has passed since the last spike. This inaccuracy in the P^*^ term is then further amplified when the weights and intrinsic excitabilities are calculated using (Equation 5) as for small values of *x*, log(*x*) approaches its vertical asymptote. The standard deviations visible in Figure 2 reflect the fact that for the spiking learning rule, the realization of Poisson noise that determined firing rates was different for each trial, but with a rate modulation that was repeated across trials.

### 3.2. Learning sequential attractors using spike-based BCPNN

**Figure 3:**
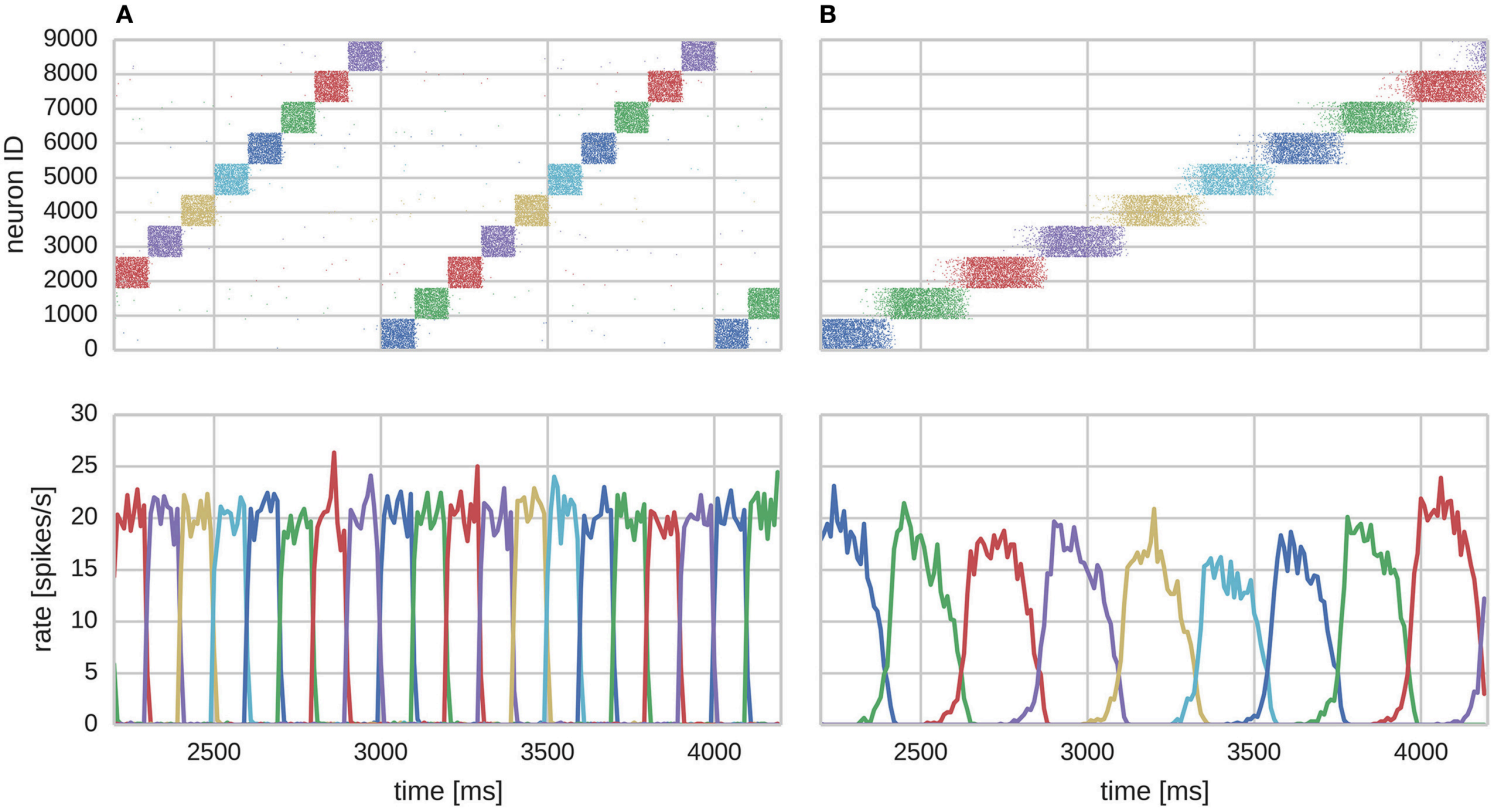
**Learning sequential attractor states**. **(A)** Training. **(B)** Replay.

In this section we consider a functional use case of the the modular attractor network described in Section 2.1 involving learning temporal sequences of attractors. With asymmetrical BCPNN time constants, it was previously proposed that this network could self-organize spontaneously active sequential attractor trajectories (Tully et al., 2014). We built a suitable network using the neuron and plasticity models described in Sections 2.2, 2.3; and the parameters listed in Table 2. Using this network we employed a training regime—a subset of which is shown in Figure 3A—in which we repeatedly stimulated all cells in a mutually exclusive sequence of minicolumns for 50 training epochs. Each minicolumn was stimulated for 100 ms, such that the neurons within it fired at an average rate of *f*_*max*_ Hz. During training we disabled the term in Equation (8) that incorporates input from the plastic AMPA and NMDA synapses meaning that, while the weights were learned online, the dynamics of the network did not disturb the training regime. A recall phase followed this learning phase in which a 50 ms stimulus of *f*_*max*_ Hz was applied to all cells in the first minicolumn of the learned sequence. During both the training and recall phases we provided background input to each cell in the network from an independent 65 Hz Poisson spike source. These Poisson spike sources are simulated on additional SpiNNaker cores to those used for the neural simulation algorithm described in Section 2.4.

Table 2**Parameters for the modular attractor network**.

**Table:** 

**(A) Model summary**	
Populations and connectivity	Modular structure described in Section 2.1
Neuron model	Leaky integrate-and-fire with exponential-shaped synaptic current inputs and spike-frequency adaption (Liu and Wang, 2001)
Synapse model	Current-based with exponential-shaped PSCs
Plasticity	BCPNN AMPA and NMDA synapses
Input	Externally generated Poisson spike trains and independent fixed-rate Poisson spike trains
Measurements	Spiking activity, membrane voltages, intrinsic bias current and synaptic weights

**Table:** 

**(B) Neuron and synapse model**	
Type	Leaky integrate-and-fire with exponential-shaped synaptic current inputs and spike-frequency adaption (Liu and Wang, 2001) as described in Section 2.3
Parameters	τ_*m*_ = 20 ms membrane time constant
	*C*_*m*_ = 250 pF membrane capacitance
	*V*_*t*_ = −50 mV threshold voltage
	*V*_*r*_ = −70 mV reset voltage
	α = 0.15 nA adaption current
	τ_*a*_ = 300 ms adaption time constant
	τ_AMPA_ = 5 ms AMPA synapse time constant
	τ_GABA_ = 5 ms GABA synapse time constant
	τ_NMDA_ = 150 ms NMDA synapse time constant

**Table:** 

**(C) Plasticity**	
Type	BCPNN AMPA synapses as described in Section 2.2
Parameters	*f*_*max*_ = 20 Hz maximum spiking frequency
	τ_*z*_*i*__ = 5 ms presynaptic primary trace time constant
	τ_*z*_*j*__ = 5 ms postsynaptic primary trace time constant
	τ_*p*_ = 2000 ms probability trace time constant
	$w_{gain}^{syn}=\frac{0.546}{N_{HC}}\text{nA}$ weight gain
Type	BCPNN NMDA synapses as described in Section 2.2
Parameters	*f*_*max*_ = 20 Hz maximum spiking frequency
	τ_*z*_*i*__ = 5 ms presynaptic primary trace time constant
	τ_*z*_*j*__ = 150 ms postsynapticprimary trace time constant
	τ_*p*_ = 2000 ms probability trace time constant
	$w_{gain}^{syn}=\frac{0.114}{N_{HC}}\text{nA}$ weight gain
	β_*gain*_ = 0.05 nA intrinsic bias gain

**Table:** 

**(D) Input**	
**Type**	**Description**
Externally generated Poisson spike trains	As described in Section 3.2
Independent fixed-rate Poisson spike trains	As described in Section 2.1

*After Nordlie et al. (2009)*.

We found that the training regime was able to produce the recurrent connectivity required to perform temporal sequence recall in the same serial order that patterns were presented during training as shown in Figure 3B. Each sequence element replayed as a learned attractor state that temporarily stifled the activity of all other cells in the network due to WTA and asymmetrically projected NMDA toward neurons of the subsequent sequence element, allowing a stable trajectory to form. Activity within attractor states was sharpened and stabilized by learned auto-associative AMPA connectivity; and sequential transitions were jointly enabled by neural adaptation and inter-pattern heteroassociation via NMDA synapses.

**Figure 4:**
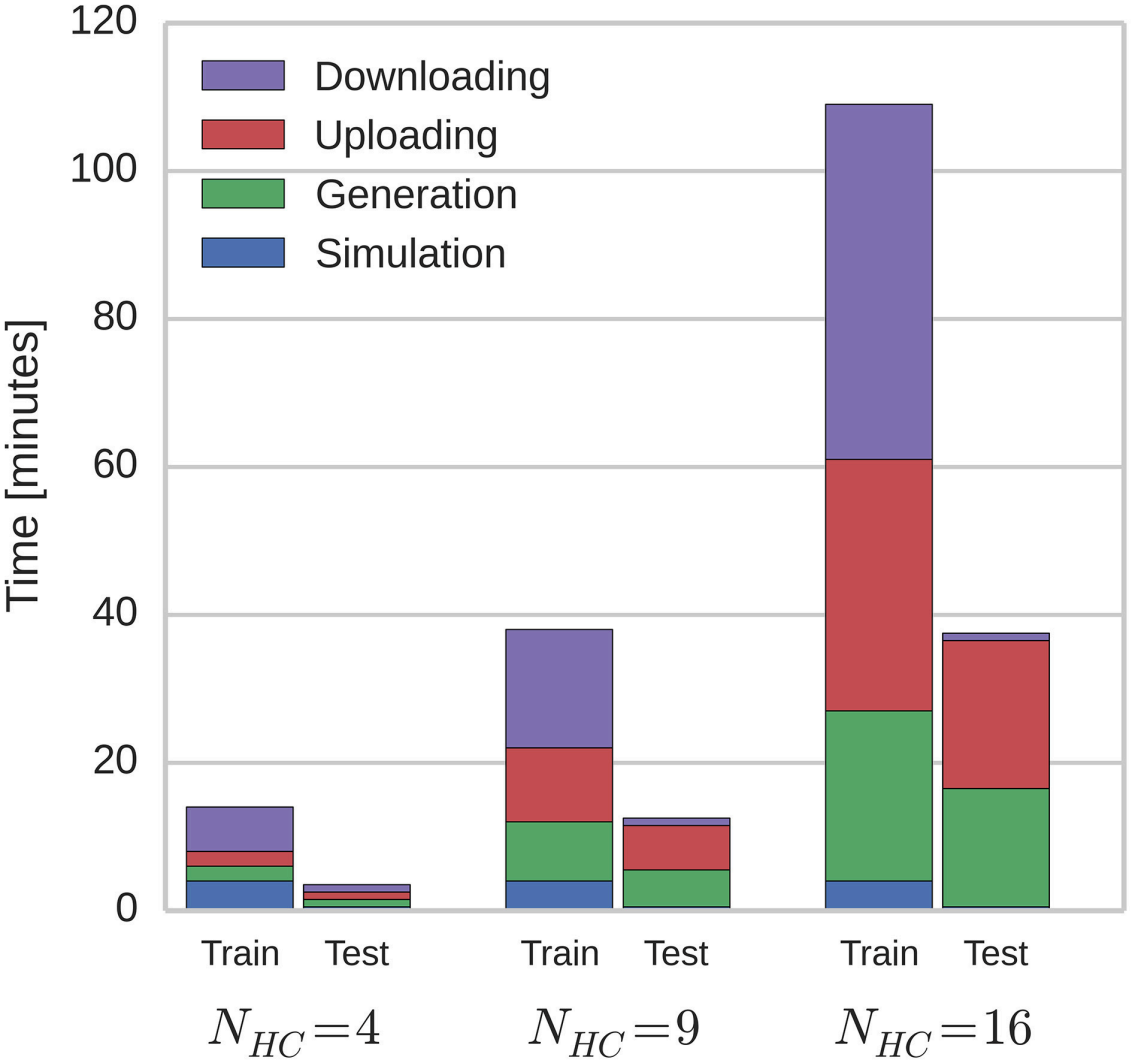
**Total simulation time on SpiNNaker**.

Because of the modular structure of the network described in Section 2.1, this temporal sequence learning can be performed using networks of varying scales by instantiating different number of hypercolumns and linearly scaling the $w_{gain}^{syn}$ parameter of the connections between them. By doing this, we investigated how the time taken to simulate the network on SpiNNaker scales with network size. Figure 4 shows how these times are split between the training and testing phases; and how long is spent generating data on the host computer, transferring it to and from SpiNNaker and actually running the simulation. As the SpiNNaker simulation always runs at a fixed fraction of real-time (for this simulation 0.5×), the simulation time remains constant as the network grows, but the times required to generate the data and to transfer it grow significantly, meaning that when *N*_*HC*_ = 16 (2.0 × 104 neurons and 5.1 × 107 plastic synapses), the total simulation time is 146 min. However, the amount of time spent in several phases of the simulation is increased by limitations of the current SpiNNaker toolchain. 84 min is spent downloading the learned weight matrices and re-uploading them for the testing: A process that is only required because the changing of parameters (in this case, whether learning is enabled or not) mid-simulation is not currently supported. Additionally, the current implementation of the algorithm outlined in Section 2.4 only allows neurons simulated on one core to have afferent synapses with a single learning rule configuration. This means that we have to run the training regime twice with the same input spike trains, once for the AMPA synapses and once for the NMDA synapses: Doubling the time taken to simulate the training network.

Previous supercomputer simulations of modular attractor memory networks have often used more complex neuron models and connectivity (Lundqvist et al., 2010), making simulation times difficult to compare with our SpiNNaker simulation due to the simplifications we outlined in Section 2.1. In order to present a better comparison, we built a network model with the same connectivity as our SpiNNaker model and simulated it on a Cray XC-30 supercomputer system using NEST version 2.2 (Gewaltig and Diesmann, 2007) with the spike-based BCPNN implementation developed by Tully et al. (2014). NEST does not include the adaptive neuron model we described in Section 2.3 so we used the adaptive exponential model (Brette and Gerstner, 2005): a simple point neuron model with spike-frequency adaption.

As previously discussed SpiNNaker runs at a fixed-fraction of real-time so we distribute our NEST simulations across increasing numbers of Cray XC-30 compute nodes (each consisting of two 2.5 GHz Intel Ivy Bridge Xeon processors) until the simulation completed in the same time as those shown in Figure 4 for our SpiNNaker simulations. Table 3 shows the result of both these supercomputer simulations and a second set with the time taken for the mid-simulation downloading and re-uploading of weights—currently required by the SpiNNaker software—removed. Due to this redundant step and because NEST parallelizes the generation of simulation data across the compute nodes, at all three scales, our modular attractor network can be simulated using 2 compute nodes. However, if we remove the time spent downloading and re-uploading the weights, 9 compute nodes are required to match the run-time of the SpiNNaker simulation when *N*_*HC*_ = 16.

**Table 3:** **Comparison of power usage of modular attractor network simulations running on SpiNNaker with simulations distributed across enough compute nodes of a Cray XC-30 system to match SpiNNaker simulation time**.

**Simulation**		**SpiNNaker**		**Cray XC-30**	
***N*_*HC*_**	**time [min]**	**# chips**	**Peak CPU power usage [W]**	**# compute nodes**	**Peak CPU power usage [W]**
4	17	6	6	2	938
9	50	12	12	2	938
16	146	21	21	2	938
4	9	6	6	4	1875
9	23	12	12	14^a^	6563
16	62	21	21	9	4219

*Cray XC-30 power usage is based on the 30 kW power usage of an entire Cray XC-30 compute rack (Cray, 2013). SpiNNaker power usage is based on the 1 W peak power usage of the SpiNNaker chip (Furber et al., 2014)*.

*Top: SpiNNaker simulation times include downloading of learned weights and re-uploading required by current software*.

*Bottom: Time taken to download learned weights, re-generate and re-upload model to SpiNNaker have been removed*.

a *We are unsure why more supercomputer compute nodes are required to match the SpiNNaker simulation times when *N*_*HC*_ = 9 than when *N*_*HC*_ = 16. We assume this is an artifact of the different scaling properties of the two simulators, but further investigation is outside of the scope of this work*.

While a more in-depth measurement of power usage is out of the scope of this work, we can also derive approximate figures for the power usage of our simulations running on both systems based on the 1 W peak power usage of the SpiNNaker chip and the 30 kW power usage of a Cray XC-30 compute rack (Cray, 2013). While these figures ignore the power consumed by the host computer connected to the SpiNNaker system; the power consumed by the “blower” and storage cabinets connected to the Cray XC-30; and assume that all CPUs are running at peak power usage, they show that even in the worst case, SpiNNaker uses 45× less power than the Cray XC-30 and, if the limitations of the current SpiNNaker software are addressed, this can be improved to 200×.

### 3.3. Connectivity patterns show different signatures in membrane potentials

**Figure 5:**
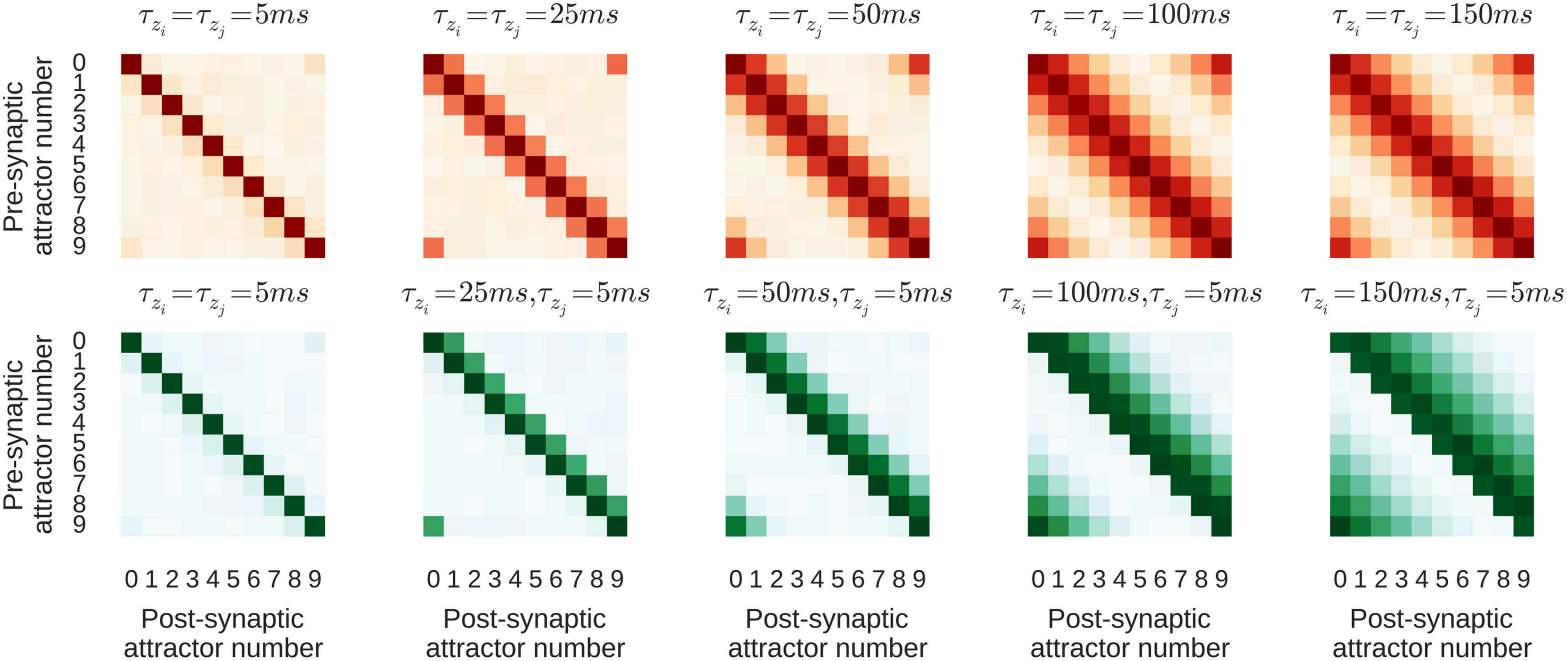
**Average strength of NMDA connections between attractors resulting from different learning time constants**. Darker colors correspond to larger synaptic weights. τ_*z*_*i*__ increases from left-to-right. Top, red row: Symmetrical kernel with τ_*z*_*j*__ = τ_*z*_*i*__. Bottom, green row: Asymmetrical kernel with τ_*z*_*j*__ = 5 ms.

The purpose of this section is to study how learning parameters influence the resulting connectivity patterns and the effect of learned connectivity on membrane dynamics during sequence replay. For this purpose we vary two parameters of the learning rule that control the time window within which correlations are detected − τ_*z*_*i*__ on the pre- and τ_*z*_*j*__ on the postsynaptic side. The network is trained using the same regime described in Section 3.2 and two different configurations, one with τ_*z*_*i*__ = τ_*z*_*j*__ on NMDA synapses, and one with τ_*z*_*i*__ ≠ τ_*z*_*j*__. If τ_*z*_*i*__ and τ_*z*_*j*__ are equal, the *Z*_*i*_ and *Z*_*j*_ traces evolve in the same manner, meaning that, as their dynamics propagate through the *P* traces to the synaptic weights, the forward and reciprocal connections between minicolumns develop symmetrically as shown in the top row of Figure 5. However, when τ_*z*_*i*__ ≠ τ_*z*_*j*__, the *Z*_*i*_ and *Z*_*j*_ traces evolve differently and, as the bottom row of Figure 5 shows, asymmetrical connections develop between minicolumns. It is important to note that the spiking activity during the training regime is the same in both configurations and the shape of the resulting connectivity results only from the learning time-constants τ_*z*_*i*__ and τ_*z*_*j*__.

**Figure 6:**
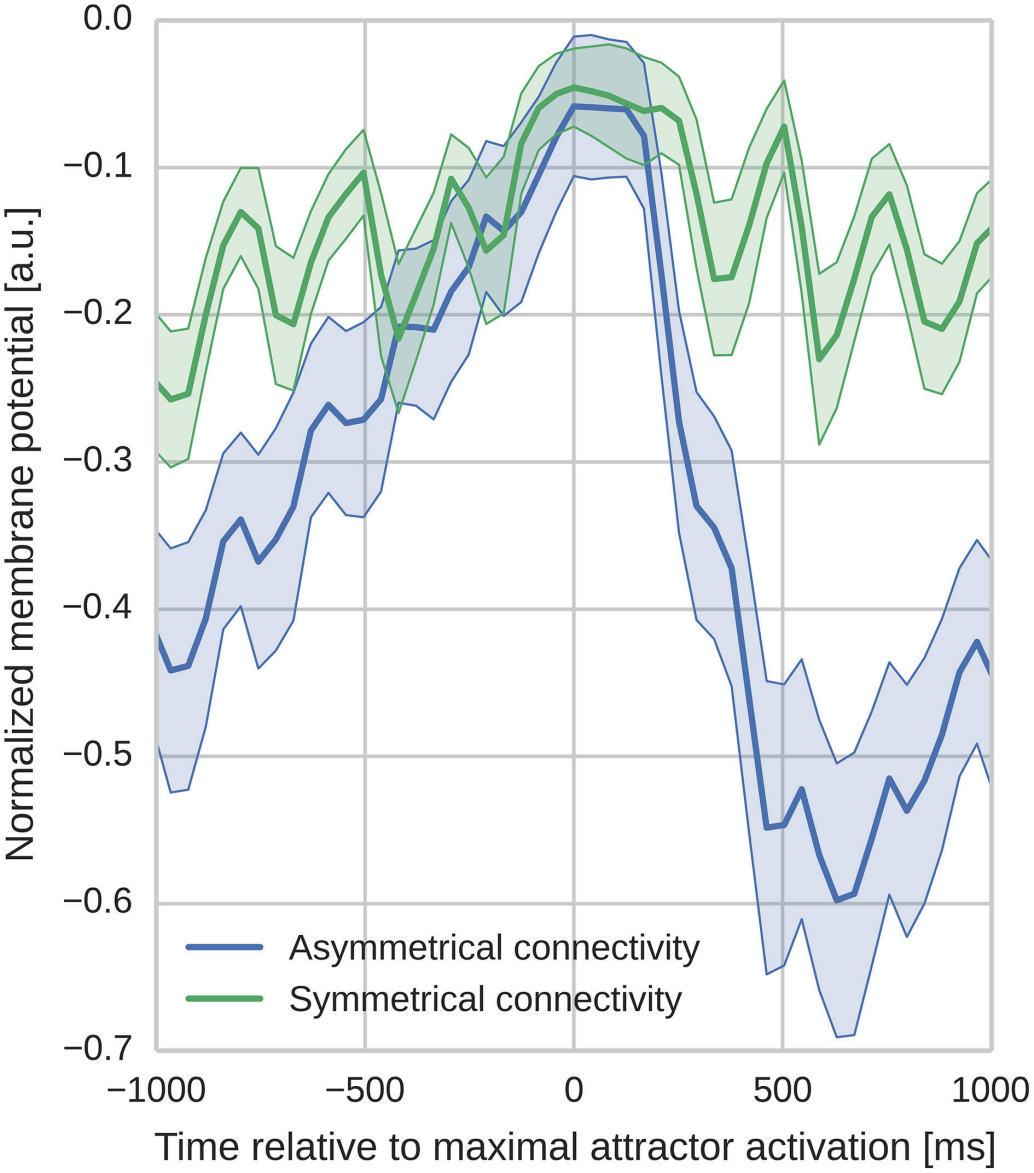
**Aligned average membrane potentials during sequence replay for two different connectivities**. The membrane potentials have been recorded from all neurons in the trained network during sequence replay. These membrane voltages have then been averaged and aligned to the time of peak activity in the temporal domain (bold lines represent the mean, shaded areas represent the standard deviation). The y-axis has been normalized to improve visibility (0 corresponds to *V*_*t*_ and −1 corresponds to the minimal membrane voltage in the sample). In the network with asymmetric connectivity the mean membrane response shows a pronounced drop after the peak response, whereas the network with symmetric connectivity does not. Oscillatory behavior originates from switches between discrete attractor states alternated by phases of inhibitory feedback.

In order to analyze the effect of the different learned connectivity patterns shown in Figure 5, we studied the impact of the two connectivity kernels on the subthreshold dynamics of neurons during sequence replay. As described in Section 3.2, after training, the trained sequence can be replayed by applying a 50 ms stimulus of *f*_*max*_ Hz into all cells of the first minicolumn in the learned sequence. Later, in the sequence replay when the stimulus has been removed, we recorded the membrane potential of all of the neurons in the network and stored the point in time when the firing rate of the respective minicolumn was maximal. We then align the membrane potential traces to this point in time and average them over all cells in a minicolumn. Interestingly, as Figure 6 illustrates, these averaged and aligned membrane responses show different characteristics for the network models built on symmetric and asymmetric connectivity. Both network types show similar membrane characteristics before the sequence arrives at the minicolumn, but, the network with symmetric connectivity shows a significantly slower decrease in membrane potential after the sequence has passed. In contrast, the network with asymmetric connectivity shows a strong after-stimulus hyperpolarization due to the increased inhibitory input originating from minicolumns later in the sequence which get subsequently activated. The slower decrease in the mean membrane potential in the symmetric network can be explained by the excitatory projections in both directions of the sequence providing excitatory current flow to previously activated neurons. The implications of this experiment and interpretations of these different characteristics is discussed in Section 4.2.

## 4. Discussion

The contribution of this study is threefold. Firstly, we have shown that BCPNN can be efficiently implemented within the constraints of the SpiNNaker neuromorphic architecture. Secondly, we have shown how BCPNN can be used in a functionally meaningful context to perform near real-time learning of temporal sequences within a large-scale modular attractor network—the largest plastic neural network ever to be simulated on neuromorphic hardware. Finally, we have demonstrated the value of SpiNNaker as a tool for investigating plasticity within large-scale brain models by exploring how, by changing a single parameter in the BCPNN learning rule, both symmetric and asymmetric connectivity can be learned, which in turn influence underlying membrane potential characteristics.

### 4.1. Learning temporal sequences in cortical microcircuits

The total duration of temporal sequences is longer than the time courses of individual cellular or synaptic processes and therefore, such sequences are thought to be driven by circuit-level phenomena although the intricacies of this relationship have yet to be fully explored. The massively recurrent and long-range nature of cortical connectivity, taken together with the emergence of temporal sequences at fine scales and distributed over spatial areas, suggests the presence of generic cortical microcircuit mechanisms. The model presented here is modularly organized into hypercolumns, each implementing WTA dynamics (Douglas and Martin, 2004). This modular structure also allowed us to vary the number of hypercolumns the network contained without effecting its functionality (Djurfeldt et al., 2008). Such distributed systems generally exhibit an improved signal-to-noise ratio, error resilience, generalizability and a structure suitable for Bayesian calculations (McClelland et al., 1986; Barlow, 2001). Like their uniformly interconnected counterparts, they can also exhibit high variability in their spike train statistics (Litwin-Kumar and Doiron, 2012; Lundqvist et al., 2012). Moreover, due to their capacity to exhibit a rich repertoire of behaviorally relevant activity states, these topologies are also well suited for information processing and stimulus sensitivity (Lundqvist et al., 2010; Wang et al., 2011).

Previous investigations have shown that the attractors which emerge within such modular networks reproduce features of local UP states (Lundqvist et al., 2006). This observation remains consistent with the extension considered here since, *in vivo*, UP state onsets are accompanied by the sequential activation of cortical neurons (Luczak et al., 2007). This redundant neural coding scheme should not necessarily be viewed in terms of anatomical columns, but rather functional columns consisting of subgroups of neurons with similar receptive fields that are highly connected (Yoshimura et al., 2005) and co-active (Cossart et al., 2003). Similar stereotypical architectures have been used as building blocks for other unified mathematical frameworks of the neocortex (Johansson and Lansner, 2007; George and Hawkins, 2009; Bastos et al., 2012).

The dynamics of the model consists of attractors, whose activations produce self-sustaining spiking among groups of neurons spread across different hypercolumns. Activity within attractors is sharpened by the fast dynamics of the AMPA receptor, until the network transitions to a subsequent attractor due to neural adaptation and asymmetrical NMDA connectivity, both of which have longer time constants of activation. In this work we have shown how these dynamics could be learned using BCPNN, a learning rule which offers an alternative to phenomenological STDP rules that often require complementary mechanisms due to their prevailing instability (Kempter et al. 2001; Babadi and Abbott 2010; but see Gütig et al. 2003).

### 4.2. Sequence anticipation and asymmetric connectivity as observed in the membrane potential dynamics

In both the symmetric and asymmetric networks, the stimulus-aligned mean membrane potential traces show a similar rise prior to sequence arrival which can be interpreted as a form of anticipation of the impending activity peak. By anticipation we mean the premature build-up of a neuronal response which is becoming increasingly similar to the response when the actual stimulus is present and represents the neural signature of expectation or prediction of future input. Anticipation is an important function of neural circuits and is observed not only in early sensory structures such as the retina (Berry et al., 1999; Hosoya et al., 2005; Vaney et al., 2012), but also in downstream structures and cortical areas (Rao and Ballard, 1999; Enns and Lleras, 2008) which are involved in more abstract cognitive tasks (Riegler, 2001; Butz et al., 2003). Anticipation can also be regarded as a form of prediction of future events: something which Bar (2007) and Bubic et al. (2010) argue is a fundamental function of the brain. This predictive capability can improve sensory perception (Yoshida and Katz, 2011; Rohenkohl et al., 2012) and is important for other modalities such as motor control and learning (Shadmehr et al., 2010; Schlerf et al., 2012). However, the connectivity which implements this predictive or anticipatory function, and the mechanisms which give rise to it, are not well understood. We believe that BCPNN learning helps fill this gap—as we discussed in Section 3.2, it can learn functional connectivity at a network scale and, as previously argued in this section, it exhibits anticipatory behavior.

We studied the network response by looking at the membrane potential dynamics prior to and after a stimulus and compared the response of two network connectivities trained with different learning parameters. As membrane potential dynamics are the result of a multitude of parameters, we constructed identical experimental settings in terms of input, to make sure that the differences in the membrane potential dynamics can be linked as closely as possible to the differences in the underlying connectivity. That is, the only major difference between the two settings is the characteristic shape of the connectivity (either being symmetric or asymmetric, see Figure 5) resulting from different learning parameters. Since the two models implement a different flow of recurrent excitation, the gain parameters in both networks have been adjusted so that both operate in a similar activity regime in order to enable a meaningful comparison of the temporal characteristics introduced by the connectivity shape. The voltage traces arising from the different network connectivities shown in Figure 6 exhibit different post-stimulus characteristics during sequence replay, with a faster hyperpolarization happening in networks with asymmetric connectivity. Hence we propose that by aligning the average membrane voltage of a population of neurons—in a perceptual context, to its preferred stimulus and, in a task-related context, to its peak activity—and then analyzing the post-stimulus characteristics of this average voltage, the population's afferent connectivity can be inferred.

### 4.3. Asymmetric connectivity supports motion preference

In the context of visual perception of motion, asymmetric connectivity has been found to play an important role in direction-sensitive ganglion cells in the retina (Kim et al., 2008; Vaney et al., 2012). A previous study by Kaplan et al. (2013) proposed asymmetric connectivity as a means of extrapolating the trajectory of a moving stimulus in the absence of a stimulus: similar, in many respects, to the experiment presented here. Recently, this hypothesized tuning property-based connectivity has been confirmed by the observation of neuronal modules in mouse V1 that exhibit similar motion direction preference (Wertz et al., 2015). The model we present here uses a Hebbian-Bayesian rule to explain how such feature-selective connectivity between neurons tuned to similar stimulus features could arise. It could therefore serve not only as a framework for modeling observed connectivity patterns and helping to understand their functional implications, but also as a means of linking experimentally observed connectivity with earlier modeling studies (Kaplan et al., 2013) by explaining how asymmetric connectivity can arise through learning.

The question of how a preferred sequence direction could be learned and replayed is not only relevant for sensory systems, but also other systems where sequence learning, generation and replay are important (Luczak et al., 2015). We addressed this question by training networks with both symmetric and asymmetric connectivity using a single sequence direction. We then triggered sequence replay in both networks in a similar way to experiments by Gavornik and Bear (2014) and Xu et al. (2012) which studied sequence learning in early visual cortices. Models with symmetric connectivity can show sequence replay in both directions, not only in the trained one. The intuition being that if one were to employ the same training protocol described in Section 3.2, one could replay the sequence forwards or backwards by presenting a cue to the first or last attractor. Instead of being directed by asymmetrical connectivity, the preferred sequence trajectory would evolve according to adaptation. Hence, the direction of the sequence during training alone is not sufficient to create a preferred replay direction as observed in experiments (Xu et al., 2012). Instead, we argue that the asymmetric connectivity caused by a difference in the learning parameters, i.e., an unequal temporal correlation time window, is necessary to replay sequences in only the trained, and therefore preferred, direction.

### 4.4. Results in context of anatomical data

The presented model addresses the question of how connectivity emerges at a cellular and network level in response to temporally varying stimuli. Through usage of different learning time constants, connectivity kernels of varying widths develop as shown in Figure 5. There exists a large body of anatomical evidence reporting regional variations in cortical circuitry in terms of structural features such as dendritic morphology and the density of dendritic spines (see e.g., Jacobs and Scheibel, 2002; Elston, 2003 for reviews). In the visual system the hierarchical organization of areas (Riesenhuber and Poggio, 1999) is reflected in their varying dendritic complexity. When compared to areas such as V1, V2, and V4 which respond to simpler visual features, areas associated with more complex functionality also exhibit more complex dendritic morphologies and have a higher number of connections (Jacobs and Scheibel, 2002; Elston and Fujita, 2014).

It stands to reason that the structural and electrophysiological differences observed in both pyramidal cells and interneurons influences activity on a cellular level (Spruston, 2008), shaping the way in which information is integrated and therefore the functional roles of both the individual cells and the entire circuit (Elston, 2003). These regional variations appear to be consistent across species and to change during development (see Elston and Fujita, 2014 for a recent review). Pyramidal cells in V1 reduce their dendritic complexity and those in inferotemporal and prefrontal areas grow larger dendritic structures over the first months and years of development. In the light of the presented model, these observations could lead to the interpretation that reducing the dendritic extent of pyramidal cells mirrors an improved perceptual precision in V1 as finer temporal correlations are detected (represented by short learning time constants τ_*z*_*i, j*__ and smaller dendritic extent as shown in the panels on the left side of Figure 5). In contrast, as more abstract associations are learned, pyramidal cells in higher areas grow more spines over larger dendritic territories. This allows these cells to integrate information from more diverse sources, requiring integration and association to occur over longer time scales (larger τ_*z*_*i, j*__). In this context, it is important to note that the learning time constants may not necessarily equal the synaptic time constants (which are determined by receptor and channel kinetics), but could vary depending on the area and with it the function or task to be learned.

Despite the fact that our model uses point neurons and thus does not directly represent the dendritic field, we argue that the learning time-constants determine a neuron's capability to integrate information over time which – given a topographic stimulus representation such as that seen in V1—could be linked to the size of the dendritic field of a neuron. Hence, the presented learning framework offers the possibility to study these arguments in more quantitative detail.

### 4.5. Scaling the modular attractor model

In Section 3.2 we presented simulations of the modular attractor network model described in Section 2.1 with up to 16 hypercolumns, connected using sparse, random 10 % global connectivity. At this scale each pyramidal cell in the network receives 4.0 × 103 afferent excitatory synapses but—if the model were scaled up to, for example, the scale of the mouse neocortex with approximately 1.6 × 107 neurons (Braitenberg and Schüz, 2013)—each pyramidal cell would receive 1.3 × 106 afferent synapses. As we discussed in Section 4.4, pyramidal cell connectivity varies widely across the layers and areas of the neocortex. However, in this section we base our discussion of the scaling properties of our model on the assumption that each pyramidal cell receives 8.0 × 103 afferent synapses. This number is consistent with averages calculated across cortical layers and areas in mice (Braitenberg and Schüz, 2013), cats (Beaulieu and Colonnier, 1989) and humans (Pakkenberg et al., 2003). The reason this number is significantly lower than the one obtained by naïvely scaling our current model is because of the “patchy” nature of long-range cortical connectivity (Goldman and Nauta, 1977; DeFelipe et al., 1986; Gilbert and Wiesel, 1989; Bosking et al., 1997). Specifically, each pyramidal cell only connects to around 10, approximately hypercolumn-sized, clusters of neurons located within a radius of a few millimeters. Additionally, while each hypercolumn in our model contains 10 minicolumns, biological hypercolumns typically have closer to 100 (Mountcastle, 1997; Buxhoeveden and Casanova, 2002). This means that, because of the winner-take-all dynamics within each hypercolumn, while 10 % of neurons in our model are active at any given time, only 1 % would be active in a more realistic model.

As Sharp and Furber (2013) discuss, when simulating spiking neural networks on SpiNNaker, the majority of CPU time is spent within the event-driven synaptic processing stage, making the CPU load highly dependent on the rate of incoming *synaptic events* (a single spike innervating a single synapse). The combined effect of the more realistic global connectivity and sparser activity discussed in the previous paragraph would be to reduce the rate of incoming synaptic events by a factor of 5 when compared to our current model. This means that a model with more realistic connectivity could actually run faster than the current model on SpiNNaker - Potentially in biological real-time rather than the 0.5× real-time we use in this work.

However, as we discussed in Section 3.2, the time spent actually running simulations on SpiNNaker is often dwarved by the time spent generating simulation data on the host computer and transferring it to and from the SpiNNaker system. One way of reducing the time taken to generate the simulation data and upload it to SpiNNaker would be to perform some of the data generation on SpiNNaker itself. The most obvious target for this approach would be the generation of the connectivity matrices as, not only do these represent the bulk of the uploaded data, but they are typically defined probabilistically meaning that they could be generated based on a very small uploaded definition. While this approach would undoubtedly reduce the time taken to generate and upload the simulation data, even the 1 min currently taken to download the results at the end of the simulation would grow to several hours if the network was scaled up to the size of even a mouse's neocortex. These slow upload and download times are due to current simulations all having been run on single board SpiNNaker systems, connected to the host computer through a single ethernet link. While the theoretical bandwidth of this link is 100 Mbit s-1, inefficiencies in the current SpiNNaker system software reduce the effective bandwidth to only a few MiB s-1.

Not only is work currently underway to improve the bandwidth of the ethernet links, but in the case of large-scale network simulations running across multiple SpiNNaker boards, if the host computer is powerful enough and connected to the SpiNNaker system through a sufficiently fast network, data can be transferred to multiple SpiNNaker boards in parallel. Furthermore, if still more bandwidth is required, each SpiNNaker board also has several high-speed serial connectors which could be used for transferring data to and from SpiNNaker at the full 1 Gbit s-1 bandwidth of the chip-level interconnect network. Together, the improvements to the scalability of the model discussed in this section would also act to further increase the power efficiency of SpiNNaker when compared to traditional super computer systems that we briefly discuss in Section 3.2.

### 4.6. Extensions of BCPNN on SpiNNaker and other future considerations

Since we have shown that BCPNN learning is possible on SpiNNaker, the implementation we describe in Section 2.5 could be extended to support spike-based reinforcement learning (Izhikevich, 2007) by adding an extra level of *E* (i.e., “eligibility”) traces with time constants between those of the *Z* and *P* traces (Tully et al., 2014). Representing downstream cellular processes that interact with increased intracellular Ca^2+^ concentrations (Yagishita et al., 2014), *E* traces propagate into the *P* traces at a rate previously described as κ (Tully et al., 2014). The κ parameter models the delivery of delayed reward signals in the form of interactions with global neuromodulatory systems, which have been linked to the emergence of sequential activity (Gavornik and Bear, 2014; Ikegaya, 2004). Using this extended BCPNN model, the modular attractor memory model we describe in Section 2.1 could be extended to include basal ganglia input (Berthet et al., 2012), allowing it to switch between behavioral sequences when this might be a beneficial strategy for successful task completion (Ponzi and Wickens, 2010).

Similarly, Vogginger et al.'s (2015) original event-driven BCPNN model includes a set of E^*^ state variables which are used to represent the components of the spike-response model arising from *E* trace dynamics. Though omitted here, the SpiNNaker BCPNN implementation could be extended to include these traces at the cost of some extra computational cost, and the memory required to store an additional 16 bit trace with each synapse and with each entry in the postsynaptic history structure. In Section 3.1 we showed that by using a 16 bit fixed-point representation for the Z^*^ and P^*^ state variables, we can produce results comparable to previous floating-point implementations when both τ_*p*_ and *f*_*max*_ are relatively small. However, this approach doesn't scale to the type of model described by Fiebig and Lansner (2014) where learning time constants span many orders of magnitude. In these situations, it may be necessary to use a 32 bit fixed-point representation for the P^*^ traces, further increasing the memory and computational cost of the learning rule.

As spikes from neuromodulator-releasing populations can arrive at the synapse at any time, integrating spike-based reinforcement learning into an event-driven, distributed simulation requires incorporating the times of modulatory as well as postsynaptic spikes into algorithm 1. Because entire populations of neuromodulator-releasing neurons can affect the modulatory input received by a single synapse, the per-neuron history structure discussed in Section 2.4 is not a viable means of storing them. Potjans et al. (2010) extend Morrison et al.'s (2007) STDP algorithm to support neuromodulated learning by introducing “volume transmitter” populations which handle all the incoming modulatory input to a virtual “volume.” These populations maintain a spike-history of all incoming modulatory spikes which they deliver to the synapses of neuronal populations within this volume, both at presynaptic spike times and after a fixed period so as to ‘flush out’ the spike-history data structure and allow it to be kept relatively small. This approach has the potential to map well to the SpiNNaker architecture and could be used as the basis of a future SpiNNaker implementation of spike-based reinforcement learning using BCPNN.

A benefit of the model proposed here is its robustness and flexibility. Non-sequential attractor networks without learning have previously been emulated on a neuromorphic microchip (Pfeil et al., 2013) and on a simulated version of the BrainScaleS system (Petrovici et al., 2014). Though not shown here, the connectivity required by these types of randomly hopping attractor networks can also be learned. Variations of this network run on supercomputers have been shown to account for disparate cognitive phenomena including perceptual rivalry and completion (Kaplan and Lansner, 2013); attentional blink (Lundqvist et al., 2006; Silverstein and Lansner, 2011); and diverse oscillatory regimes (Lundqvist et al., 2010). But our model was a reduced version of previous detailed ones insofar that we did not utilize Hodgkin-Huxley neurons with calcium-dependent potassium channels or regular spiking non-pyramidal cells; nor did we explicitly model connections among basket cells, saturating synapses, a Vm-dependent Mg^2+^ blockade or short-term depression.

A problem not stressed by the aforementioned models is how the connectivity required for stable activity propagation might be learned (Wörgötter and Porr, 2005; Kunkel et al., 2011), despite the biochemical (Peters et al., 2014) and metabolic (Picard et al., 2013) changes accompanying learned sequential behaviors. Several promising approaches have been developed (Sussillo and Abbott, 2009; Laje and Buonomano, 2013; Hennequin et al., 2014), albeit with biological motivations driven more from the perspective of algorithmic optimization, rather than from bottom-up neural processing. Here, we have shown that activity could propagate through recurrent cortical microcircuits as a result of a probabilistic learning rule based on neurobiologically plausible time courses and dynamics. The model predicts that the interaction between several learning and dynamical processes constitute a compound mnemonic engram that can flexibly generate step-wise sequential increases of activity within pools of excitatory neurons. We have shown that this large-scale learning model can be efficiently simulated at scale using neuromorphic hardware and our simulations suggest that flexible systems such as SpiNNaker offer a promising tool for the study of collective dynamical phenomena emerging from the complex interactions occurring between individual neurons and synapses whose properties change over time.

## Author contributions

JK developed the SpiNNaker BCPNN implementation and performed the experiments. JK and BK analyzed the data. PT, AL, and JK developed the simplified modular attractor network architecture. JK, PT, BK, AL, and SF wrote the paper.

### Conflict of interest statement

The authors declare that the research was conducted in the absence of any commercial or financial relationships that could be construed as a potential conflict of interest.

## References

[B1] Abbott L. F. Blum K. I. (1996). Functional significance of long-term potentiation for sequence learning and prediction. Cereb. Cortex 6, 406–416. 10.1093/cercor/6.3.406 8670667

[B2] Abeles M. Bergman H. Gat I. Meilijson I. Seidemann E. Tishby N.. (1995). Cortical activity flips among quasi-stationary states. Proc. Natl. Acad. Sci. U.S.A. 92, 8616–8620. 10.1073/pnas.92.19.8616 7567985 PMC41017

[B3] Babadi B. Abbott L. F. (2010). Intrinsic stability of temporally shifted spike-timing dependent plasticity. PLoS Comput. Biol. 6:e1000961. 10.1371/journal.pcbi.1000961 21079671 PMC2973812

[B4] Bar M. (2007). The proactive brain: using analogies and associations to generate predictions. Trends Cogn. Sci. 11, 280–289. 10.1016/j.tics.2007.05.005 17548232

[B5] Barlow H. (2001). Redundancy reduction revisited. Network 12, 241–253. 10.1080/net.12.3.241.253 11563528

[B6] Bastos A. M. Usrey W. M. Adams R. A. Mangun G. R. Fries P. Friston K. J. (2012). Canonical microcircuits for predictive coding. Neuron 76, 695–711. 10.1016/j.neuron.2012.10.038 23177956 PMC3777738

[B7] Beaulieu C. Colonnier M. (1989). Number and size of neurons and synapses in the motor cortex of cats raised in different environmental complexities. J. Comp. Neurol. 289, 178–187. 10.1002/cne.902890115 2808760

[B8] Benjamin B. V. Gao P. McQuinn E. Choudhary S. Chandrasekaran A. R. Bussat J. M. (2014). Neurogrid: a mixed-analog-digital multichip system for large-scale neural simulations. Proc. IEEE 102, 699–716. 10.1109/JPROC.2014.2313565

[B9] Berry M. J. II, Brivanlou, I. H. Jordan T. A. Meister M. (1999). Anticipation of moving stimuli by the retina. Nature 398, 334–338. 10.1038/18678 10192333

[B10] Berthet P. Hellgren-Kotaleski J. Lansner A. (2012). Action selection performance of a reconfigurable basal ganglia inspired model with hebbian–bayesian go-nogo connectivity. Front. Behav. Neurosci. 6:65. 10.3389/fnbeh.2012.00065 23060764 PMC3462417

[B11] Bi G.-Q. Poo M.-M. (1998). Synaptic modifications in cultured hippocampal neurons: dependence on spike timing, synaptic strength, and postsynaptic cell type. J. Neurosci. 18, 10464–10472. 9852584 10.1523/JNEUROSCI.18-24-10464.1998 PMC6793365

[B12] Bosking W. H. Zhang Y. Schofield B. Fitzpatrick D. (1997). Orientation selectivity and the arrangement of horizontal connections in tree shrew striate cortex. J. Neurosci. 17, 2112–2127. 9045738 10.1523/JNEUROSCI.17-06-02112.1997 PMC6793759

[B13] Braitenberg V. Schüz A. (2013). Cortex: Statistics and Geometry of Neuronal Connectivity. Berlin; Heidelberg; New York, NY: Springer Science & Business Media.

[B14] Brette R. Gerstner W. (2005). Adaptive exponential integrate-and-fire model as an effective description of neuronal activity. J. Neurophysiol. 94, 3637–3642. 10.1152/jn.00686.2005 16014787

[B15] Bubic A. von Cramon D. Y. Schubotz R. I. (2010). Prediction, cognition and the brain. Front. Hum. Neurosci. 4:25. 10.3389/fnhum.2010.00025 20631856 PMC2904053

[B16] Butz M. V. Sigaud O. Gérard P. (2003). Internal models and anticipations in adaptive learning systems, in Anticipatory Behavior in Adaptive Learning Systems, eds Butz M. V. Sigaud O. Gérard P. (Heidelberg; Berlin: Springer), 86–109. 10.1007/978-3-540-45002-3_6

[B17] Buxhoeveden D. P. Casanova M. F. (2002). The minicolumn hypothesis in neuroscience. Brain 125, 935–951. 10.1093/brain/awf110 11960884

[B18] Caporale N. Dan Y. (2008). Spike timing-dependent plasticity: a hebbian learning rule. Annu. Rev. Neurosci. 31, 25–46. 10.1146/annurev.neuro.31.060407.125639 18275283

[B19] Cossart R. Aronov D. Yuste R. (2003). Attractor dynamics of network up states in the neocortex. Nature 423, 283–288. 10.1038/nature01614 12748641

[B20] Cray (2013). Cray XC30-ACø Supercomputer. Technical Report, Cray Inc.

[B21] Dan Y. Poo M.-M. (2004). Spike timing-dependent plasticity of neural circuits. Neuron 44, 23–30. 10.1016/j.neuron.2004.09.007 15450157

[B22] Daoudal G. Debanne D. (2003). Long-term plasticity of intrinsic excitability: learning rules and mechanisms. Learn. Mem. 10, 456–465. 10.1101/lm.64103 14657257

[B23] DeFelipe J. Conley M. Jones E. (1986). Long-range focal collateralization of axons arising from corticocortical cells in monkey sensory-motor cortex. J. Neurosci. 6, 3749–3766. 2432205 10.1523/JNEUROSCI.06-12-03749.1986 PMC6568647

[B24] Diehl P. U. Cook M. (2014). Efficient Implementation of STDP Rules on SpiNNaker Neuromorphic Hardware, in Neural Networks (IJCNN), The 2014 International Joint Conference on (Beijing), 4288–4295. 10.1109/IJCNN.2014.6889876

[B25] Djurfeldt M. Lundqvist M. Johansson C. Rehn M. Ekeberg O. Lansner A. (2008). Brain-scale simulation of the neocortex on the IBM Blue Gene/L supercomputer. IBM J. Res. Dev. 52, 31–41. 10.1147/rd.521.0031

[B26] Douglas R. J. Martin K. A. (2004). Neuronal circuits of the neocortex. Annu. Rev. Neurosci. 27, 419–451. 10.1146/annurev.neuro.27.070203.144152 15217339

[B27] Elston G. N. (2003). Cortex, cognition and the cell: new insights into the pyramidal neuron and prefrontal function. Cereb. Cortex 13, 1124–1138. 10.1093/cercor/bhg093 14576205

[B28] Elston G. N. Fujita I. (2014). Pyramidal cell development: postnatal spinogenesis, dendritic growth, axon growth, and electrophysiology. Front. Neuroanat. 8:78. 10.3389/fnana.2014.00078 25161611 PMC4130200

[B29] Enns J. T. Lleras A. (2008). What's next? new evidence for prediction in human vision. Trends Cogn. Sci. 12, 327–333. 10.1016/j.tics.2008.06.001 18684660

[B30] Feldman D. E. (2012). The spike-timing dependence of plasticity. Neuron 75, 556–571. 10.1016/j.neuron.2012.08.001 22920249 PMC3431193

[B31] Fiebig F. Lansner A. (2014). Memory consolidation from seconds to weeks : a three-stage neural memory consolidation from seconds to weeks : a three-stage neural network model with autonomous reinstatement dynamics. Front. Comput. Neurosci. 8:64. 10.3389/fncom.2014.00064 25071536 PMC4077014

[B32] Furber S. B. Galluppi F. Temple S. Plana L. A. (2014). The SpiNNaker project. Proc. IEEE 102, 652–665. 10.1109/JPROC.2014.2304638

[B33] Galluppi F. Lagorce X. Stromatias E. Pfeiffer M. Plana L. A. Furber S. B.. (2015). A framework for plasticity implementation on the SpiNNaker neural architecture. Front. Neurosci. 8:429. 10.3389/fnins.2014.00429 25653580 PMC4299433

[B34] Gavornik J. P. Bear M. F. (2014). Learned spatiotemporal sequence recognition and prediction in primary visual cortex. Nat. Neurosci. 17, 732–737. 10.1038/nn.3683 24657967 PMC4167369

[B35] George D. Hawkins J. (2009). Towards a mathematical theory of cortical micro-circuits. PLoS Comput. Biol. 5:e1000532. 10.1371/journal.pcbi.1000532 19816557 PMC2749218

[B36] Gerstner W. Kistler W. M. (2002). Spiking Neuron Models: Single Neurons, Populations, Plasticity. Cambridge: Cambridge University Press 10.1017/cbo9780511815706

[B37] Gewaltig M.-O. Diesmann M. (2007). NEST (NEural simulation tool). Scholarpedia 2:1430 10.4249/scholarpedia.1430

[B38] Gilbert C. D. Wiesel T. N. (1989). Columnar specificity of intrinsic horizontal and corticocortical connections in cat visual cortex. J. Neurosci. 9, 2432–2442. 2746337 10.1523/JNEUROSCI.09-07-02432.1989 PMC6569760

[B39] Goldman P. S. Nauta W. J. (1977). Columnar distribution of cortico-cortical fibers in the frontal association, limbic, and motor cortex of the developing rhesus monkey. Brain Res. 122, 393–413. 10.1016/0006-8993(77)90453-x 402978

[B40] Gütig R. Aharonov R. Rotter S. Sompolinsky H. (2003). Learning input correlations through nonlinear temporally asymmetric hebbian plasticity. J. Neurosci. 23, 3697–3714. 12736341 10.1523/JNEUROSCI.23-09-03697.2003 PMC6742165

[B41] Hennequin G. Vogels T. P. Gerstner W. (2014). Optimal control of transient dynamics in balanced networks supports generation of complex movements. Neuron 82, 1394–1406. 10.1016/j.neuron.2014.04.045 24945778 PMC6364799

[B42] Hopkins M. Furber S. (2015). Accuracy and efficiency in fixed-point neural ODE solvers. Neural Comput. 27, 2148–2182. 10.1162/NECO_a_00772 26313605

[B43] Hosoya T. Baccus S. A. Meister M. (2005). Dynamic predictive coding by the retina. Nature 436, 71–77. 10.1038/nature03689 16001064

[B44] Ikegaya Y. (2004). Synfire chains and cortical songs: temporal modules of cortical activity. Science 304, 559–564. 10.1126/science.1093173 15105494

[B45] Izhikevich E. M. (2007). Solving the distal reward problem through linkage of STDP and dopamine signaling. Cereb. Cortex 17, 2443–2452. 10.1093/cercor/bhl152 17220510

[B46] Jacobs B. Scheibel A. (2002). Regional dendritic variation in primate cortical pyramidal cells, in Cortical Areas: Unity Diversity, eds Schuez A. Miller R. (London; New York, NY: Tailor and Francis), 111–131. 10.1201/9780203299296.pt2

[B47] Jin X. Rast A. Galluppi F. Davies S. Furber S. (2010). Implementing spike-timing-dependent plasticity on SpiNNaker neuromorphic hardware, in The 2010 International Joint Conference on Neural Networks (IJCNN), Number 1 (Barcelona: IEEE), 1–8. 10.1109/ijcnn.2010.5596372

[B48] Johansson C. Lansner A. (2007). Towards cortex sized artificial neural systems. Neural Netw. 20, 48–61. 10.1016/j.neunet.2006.05.029 16860539

[B49] Jones L. M. Fontanini A. Sadacca B. F. Miller P. Katz D. B. (2007). Natural stimuli evoke dynamic sequences of states in sensory cortical ensembles. Proc. Natl. Acad. Sci. U.S.A. 104, 18772–18777. 10.1073/pnas.0705546104 18000059 PMC2141852

[B50] Jung S.-C. Hoffman D. A. (2009). Biphasic somatic a-type k+ channel downregulation mediates intrinsic plasticity in hippocampal ca1 pyramidal neurons. PLoS ONE 4:e6549. 10.1371/journal.pone.0006549 19662093 PMC2717216

[B51] Kaplan B. A. Lansner A. (2013). A spiking neural network model of self-organized pattern recognition in the early mammalian olfactory system. Front. Neural Circuits 8:5. 10.3389/fncir.2014.00005 24570657 PMC3916767

[B52] Kaplan B. A. Lansner A. Masson G. S. Perrinet L. U. (2013). Anisotropic connectivity implements motion-based prediction in a spiking neural network. Front. Comput. Neurosci. 7:112. 10.3389/fncom.2013.00112 24062680 PMC3775506

[B53] Kempter R. Gerstner W. van Hemmen J. L. (2001). Intrinsic stabilization of output rates by spike-based hebbian learning. Neural Comput. 13, 2709–2741. 10.1162/089976601317098501 11705408

[B54] Kim I.-J. Zhang Y. Yamagata M. Meister M. Sanes J. R. (2008). Molecular identification of a retinal cell type that responds to upward motion. Nature 452, 478–482. 10.1038/nature06739 18368118

[B55] Kunkel S. Diesmann M. Morrison A. (2011). Limits to the development of feed-forward structures in large recurrent neuronal networks. Front. Comput. Neurosci. 4:160. 10.3389/fncom.2010.00160 21415913 PMC3042733

[B56] Kunkel S. Potjans T. C. Eppler J. M. Plesser H. E. Morrison A. Diesmann M. (2012). Meeting the memory challenges of brain-scale network simulation. Front. Neuroinform. 5:35. 10.3389/fninf.2011.00035 22291636 PMC3264885

[B57] Lagorce X. Stromatias E. Galluppi F. Plana L. A. Liu S.-C. Furber S. B.. (2015). Breaking the millisecond barrier on SpiNNaker: implementing asynchronous event-based plastic models with microsecond resolution. Front. Neurosci. 9:206. 10.3389/fnins.2015.00206 26106288 PMC4458614

[B58] Laje R. Buonomano D. V. (2013). Robust timing and motor patterns by taming chaos in recurrent neural networks. Nat. Neurosci. 16, 925–933. 10.1038/nn.3405 23708144 PMC3753043

[B59] Lansner A. Ekeberg Ö. (1989). A one-layer feedback artificial neural network with a bayesian learning rule. Int. J. Neural Syst. 1, 77–87. 10.1142/S0129065789000499

[B60] Lansner A. Holst A. (1996). A higher order Bayesian neural network with spiking units. Int. J. Neural Syst. 7, 115–128. 10.1142/s0129065796000816 8823623

[B61] Lisman J. Spruston N. (2005). Postsynaptic depolarization requirements for ltp and ltd: a critique of spike timing-dependent plasticity. Nat. Neurosci. 8, 839–841. 10.1038/nn0705-839 16136666

[B62] Lisman J. Spruston N. (2010). Questions about stdp as a general model of synaptic plasticity. Front. Synaptic Neurosci. 2:140. 10.3389/fnsyn.2010.00140 21423526 PMC3059684

[B63] Litwin-Kumar A. Doiron B. (2012). Slow dynamics and high variability in balanced cortical networks with clustered connections. Nat. Neurosci. 15, 1498–1505. 10.1038/nn.3220 23001062 PMC4106684

[B64] Liu Y. H. Wang X. J. (2001). Spike-frequency adaptation of a generalized leaky integrate-and-fire model neuron. J. Comput. Neurosci. 10, 25–45. 10.1023/A:1008916026143 11316338

[B65] Luczak A. Barthó P. Marguet S. L. Buzsáki G. Harris K. D. (2007). Sequential structure of neocortical spontaneous activity *in vivo*. Proc. Natl. Acad. Sci. U.S.A. 104, 347–352. 10.1073/pnas.0605643104 17185420 PMC1765463

[B66] Luczak A. McNaughton B. L. Harris K. D. (2015). Packet-based communication in the cortex. Nat. Rev. Neurosci. 16, 745–755. 10.1038/nrn4026 26507295

[B67] Lundqvist M. Compte A. Lansner A. (2010). Bistable, irregular firing and population oscillations in a modular attractor memory network. PLoS Comput. Biol. 6:e1000803. 10.1371/journal.pcbi.1000803 20532199 PMC2880555

[B68] Lundqvist M. Herman P. Lansner A. (2012). Variability of spike firing during theta-coupled replay of memories in a simulated attractor network. Brain Res. 1434, 152–161. 10.1016/j.brainres.2011.07.055 21907326

[B69] Lundqvist M. Rehn M. Djurfeldt M. Lansner A. (2006). Attractor dynamics in a modular network model of neocortex. Network 17, 253–276. 10.1080/09548980600774619 17162614

[B70] Markram H. Gerstner W. Sjöström P. J. (2011). A history of spike-timing-dependent plasticity. Front. Synaptic Neurosci. 3:4. 10.3389/fnsyn.2011.00004 22007168 PMC3187646

[B71] McClelland J. L. Rumelhart D. E. Hinton G. E. (1986). The Appeal of Parallel Distributed Processing. Cambridge, MA: MIT Press.

[B72] Mead C. (1990). Neuromorphic electronic systems. Proc. IEEE 78, 1629–1636. 10.1109/5.58356

[B73] Merolla P. A. Arthur J. V. Alvarez-Icaza R. Cassidy A. S. Sawada J. Akopyan F. (2014). A million spiking-neuron integrated circuit with a scalable communication network and interface. Science 345, 668–673. 10.1126/science.1254642 25104385

[B74] Moise M. (2012). A Fixed Point Arithmetic Library for SpiNNaker. PhD thesis, The University of Manchester, Manchester.

[B75] Morrison A. Aertsen A. Diesmann M. (2007). Spike-timing-dependent plasticity in balanced random networks. Neural Comput. 19, 1437–1467. 10.1162/neco.2007.19.6.1437 17444756

[B76] Morrison A. Mehring C. Geisel T. Aertsen A. D. Diesmann M. (2005). Advancing the boundaries of high-connectivity network simulation with distributed computing. Neural Comput. 17, 1776–1801. 10.1162/0899766054026648 15969917

[B77] Mountcastle V. B. (1997). The columnar organization of the neocortex. Brain 120, 701–722. 10.1093/brain/120.4.701 9153131

[B78] Nordlie E. Gewaltig M. O. Plesser H. E. (2009). Towards reproducible descriptions of neuronal network models. PLoS Comput. Biol. 5:e1000456. 10.1371/journal.pcbi.1000456 19662159 PMC2713426

[B79] Painkras E. Plana L. (2013). SpiNNaker: a 1-W 18-core system-on-chip for massively-parallel neural network simulation. IEEE J. Solid-State Circ. 48, 1943–1953. 10.1109/JSSC.2013.2259038

[B80] Pakkenberg B. Pelvig D. Marner L. Bundgaard M. J. Gundersen H. J. G. Nyengaard J. R.. (2003). Aging and the human neocortex. Exp. Gerontol. 38, 95–99. 10.1016/S0531-5565(02)00151-1 12543266

[B81] Peters A. J. Chen S. X. Komiyama T. (2014). Emergence of reproducible spatiotemporal activity during motor learning. Nature 510, 263–267. 10.1038/nature13235 24805237

[B82] Petrovici M. A. Vogginger B. Müller P. Breitwieser O. Lundqvist M Muller L. (2014). Characterization and compensation of network-level anomalies in mixed-signal neuromorphic modeling platforms. PLoS ONE 9:e108590 10.1371/journal.pone.0108590 Available online at: http://journals.plos.org/plosone/article?id=10.1371/journal.pone.0108590 25303102 PMC4193761

[B83] Pfeil T. Grübl A. Jeltsch S. Müller E. Müller P. Petrovici M. A.. (2013). Six networks on a universal neuromorphic computing substrate. Front. Neurosci. 7:11. 10.3389/fnins.2013.00011 23423583 PMC3575075

[B84] Picard N. Matsuzaka Y. Strick P. L. (2013). Extended practice of a motor skill is associated with reduced metabolic activity in m1. Nat. Neurosci. 16, 1340–1347. 10.1038/nn.3477 23912947 PMC3757119

[B85] Ponzi A. Wickens J. (2010). Sequentially switching cell assemblies in random inhibitory networks of spiking neurons in the striatum. J. Neurosci. 30, 5894–5911. 10.1523/JNEUROSCI.5540-09.2010 20427650 PMC6632589

[B86] Potjans W. Morrison A. Diesmann M. (2010). Enabling functional neural circuit simulations with distributed computing of neuromodulated plasticity. Front. Comput. Neurosci. 4:141. 10.3389/fncom.2010.00141 21151370 PMC2996144

[B87] Rao R. P. Ballard D. H. (1999). Predictive coding in the visual cortex: a functional interpretation of some extra-classical receptive-field effects. Nat. Neurosci. 2, 79–87. 10.1038/4580 10195184

[B88] Rauch A. La Camera G. Lüscher H.-R. Senn W. Fusi S. (2003). Neocortical pyramidal cells respond as integrate-and-fire neurons to *in vivo*–like input currents. J. Neurophysiol. 90, 1598–1612. 10.1152/jn.00293.2003 12750422

[B89] Riegler A. (2001). The role of anticipation in cognition, in AIP Conference Proceedings (Liege: IOP Institute of Physics Publishing Ltd), 534–544. 10.1063/1.1388719

[B90] Riesenhuber M. Poggio T. (1999). Hierarchical models of object recognition in cortex. Nat. Neurosci. 2, 1019–1025. 10.1038/14819 10526343

[B91] Rohenkohl G. Cravo A. M. Wyart V. Nobre A. C. (2012). Temporal expectation improves the quality of sensory information. J. Neurosci. 32, 8424–8428. 10.1523/JNEUROSCI.0804-12.2012 22699922 PMC4235252

[B92] Sandberg A. Lansner A. Petersson K. M. Ekeberg O. (2002). A Bayesian attractor network with incremental learning. Network 13, 179–194. 10.1080/net.13.2.179.194 12061419

[B93] Schemmel J. Bruderle D. Grubl A. Hock M. Meier K. Millner S. (2010). A wafer-scale neuromorphic hardware system for large-scale neural modeling, in Circuits and Systems (ISCAS), Proceedings of 2010 IEEE International Symposium on (Paris: IEEE), 1947–1950. 10.1109/ISCAS.2010.5536970

[B94] Schlerf J. Ivry R. B. Diedrichsen J. (2012). Encoding of sensory prediction errors in the human cerebellum. J. Neurosci. 32, 4913–4922. 10.1523/JNEUROSCI.4504-11.2012 22492047 PMC4332713

[B95] Seidemann E. Meilijson I. Abeles M. Bergman H. Vaadia E. (1996). Simultaneously recorded single units in the frontal cortex go through sequences of discrete and stable states in monkeys performing a delayed localization task. J. Neurosci. 16, 752–768. 8551358 10.1523/JNEUROSCI.16-02-00752.1996 PMC6578656

[B96] Shadmehr R. Smith M. A. Krakauer J. W. (2010). Error correction, sensory prediction, and adaptation in motor control. Ann. Rev. Neurosci. 33, 89–108. 10.1146/annurev-neuro-060909-153135 20367317

[B97] Sharp T. Furber S. (2013). Correctness and performance of the SpiNNaker architecture, in Neural Networks (IJCNN), The 2013 International Joint Conference on (Dallas, TX). 10.1109/ijcnn.2013.6706988

[B98] Sharp T. Galluppi F. Rast A. Furber S. (2012). Power-efficient simulation of detailed cortical microcircuits on SpiNNaker. J. Neurosci. Methods 210, 110–118. 10.1016/j.jneumeth.2012.03.001 22465805

[B99] Sharp T. Petersen R. Furber S. (2014). Real-time million-synapse simulation of rat barrel cortex. Front. Neurosci. 8:131. 10.3389/fnins.2014.00131 24910593 PMC4038760

[B100] Sheik S. Coath M. Indiveri G. Denham S. L. Wennekers T. Chicca E. (2012). Emergent auditory feature tuning in a real-time neuromorphic VLSI system. Front. Neurosci. 6:17. 10.3389/fnins.2012.00017 22347163 PMC3272652

[B101] Silverstein D. N. Lansner A. (2011). Is attentional blink a byproduct of neocortical attractors? Front. Comput. Neurosci. 5:13. 10.3389/fncom.2011.00013 21625630 PMC3096845

[B102] Spruston N. (2008). Pyramidal neurons: dendritic structure and synaptic integration. Nat. Rev. Neurosci. 9, 206–221. 10.1038/nrn2286 18270515

[B103] Stromatias E. Galluppi F. Patterson C. Furber S. (2013). Power analysis of large-scale, real-time neural networks on SpiNNaker, in The 2013 International Joint Conference on Neural Networks (IJCNN) (Dallas, TX), 1–8. 10.1109/IJCNN.2013.6706927

[B104] Sussillo D. Abbott L. F. (2009). Generating coherent patterns of activity from chaotic neural networks. Neuron 63, 544–557. 10.1016/j.neuron.2009.07.018 19709635 PMC2756108

[B105] Tully P. J. Hennig M. H. Lansner A. (2014). Synaptic and nonsynaptic plasticity approximating probabilistic inference. Front. Synaptic Neurosci. 6:8. 10.3389/fnsyn.2014.00008 24782758 PMC3986567

[B106] Vaney D. I. Sivyer B. Taylor W. R. (2012). Direction selectivity in the retina: symmetry and asymmetry in structure and function. Nat. Rev. Neurosci. 13, 194–208. 10.1038/nrn3165 22314444

[B107] Vogginger B. Schüffny R. Lansner A. Cederström L. Partzsch J. Höppner S. (2015). Reducing the computational footprint for real-time BCPNN learning. Front. Neurosci. 9:2. 10.3389/fnins.2015.00002 25657618 PMC4302947

[B108] Wang S.-J. Hilgetag C. C. Zhou C. (2011). Sustained activity in hierarchical modular neural networks: self-organized criticality and oscillations. Front. Comput. Neurosci. 5:30. 10.3389/fncom.2011.00030 21852971 PMC3151620

[B109] Wertz A. Trenholm S. Yonehara K. Hillier D. Raics Z. Leinweber M.. (2015). Single-cell–initiated monosynaptic tracing reveals layer-specific cortical network modules. Science 349, 70–74. 10.1126/science.aab1687 26138975

[B110] Wörgötter F. Porr B. (2005). Temporal sequence learning, prediction, and control: a review of different models and their relation to biological mechanisms. Neural Comput. 17, 245–319. 10.1162/0899766053011555 15720770

[B111] Xu S. Jiang W. Poo M.-M. Dan Y. (2012). Activity recall in a visual cortical ensemble. Nat. Neurosci. 15, 449–455. 10.1038/nn.3036 22267160 PMC3288189

[B112] Yagishita S. Hayashi-Takagi A. Ellis-Davies G. C. Urakubo H. Ishii S. Kasai H. (2014). A critical time window for dopamine actions on the structural plasticity of dendritic spines. Science 345, 1616–1620. 10.1126/science.1255514 25258080 PMC4225776

[B113] Yoshida T. Katz D. B. (2011). Control of prestimulus activity related to improved sensory coding within a discrimination task. J. Neurosci. 31, 4101–4112. 10.1523/JNEUROSCI.4380-10.2011 21411651 PMC3089821

[B114] Yoshimura Y. Dantzker J. L. Callaway E. M. (2005). Excitatory cortical neurons form fine-scale functional networks. Nature 433, 868–873. 10.1038/nature03252 15729343

